# Oncology and Cardiac Rehabilitation: An Underrated Relationship

**DOI:** 10.3390/jcm9061810

**Published:** 2020-06-10

**Authors:** E. Venturini, G. Iannuzzo, A. D’Andrea, M. Pacileo, L. Tarantini, M.L. Canale, M. Gentile, G. Vitale, F.M. Sarullo, R. Vastarella, A. Di Lorenzo, C. Testa, A. Parlato, C. Vigorito, F. Giallauria

**Affiliations:** 1 Cardiac Rehabilitation Unit, Azienda USL Toscana Nord-Ovest, Cecina Civil Hospital, 57023 LI Cecina, Italy; 2 Department of Clinical Medicine and Surgery, Federico II University, 80131 Naples, Italy; gabriella.iannuzzo@unina.it (G.I.); margenti@unina.it (M.G.); 3 Unit of Cardiology and Intensive Care, “Umberto I” Hospital, Viale San Francesco, Nocera Inferiore, 84014 SA, Italy; antonellodandrea@libero.it (A.D.); pacmario@yahoo.it (M.P.); 4 Division of Cardiology, Ospedale San Martino ULSS1 Dolomiti, 32100 Belluno, Italy; luigi.tarantini@gmail.com; 5 Department of Cardiology, Azienda USL Toscana Nord-Ovest, Ospedale Versilia, Lido di Camaiore, 55041 LU, Italy; marialaura.canale@uslnordovest.toscana.it; 6 Cardiovascular Rehabilitation Unit, Buccheri La Ferla Fatebenefratelli Hospital, 90123 Palermo, Italy; giuseppevit@hotmail.com (G.V.); fsarullo@neomedia.it (F.M.S.); 7 UOSD Scompenso Cardiaco e Cardiologia Riabilitativa, AORN Ospedale dei Colli-Monaldi, 80131 Naples, Italy; rossellavastarella86@gmail.com; 8 Department of Translational Medical Sciences, Federico II University of Naples, 80131 Naples, Italy; dilorenzoanna2@gmail.com (A.D.L.); kre.testa@gmail.com (C.T.); alessandroparlato96@gmail.com (A.P.); vigorito@unina.it (C.V.); francesco.giallauria@unina.it (F.G.)

**Keywords:** cancer/cardiovascular disease bidirectional relationship, exercise therapy in cancer, protection from cancer therapy, cardiac rehabilitation, cardio-oncology rehabilitation

## Abstract

Cancer and cardiovascular diseases are globally the leading causes of mortality and morbidity. These conditions are closely related, beyond that of sharing many risk factors. The term bidirectional relationship indicates that cardiovascular diseases increase the likelihood of getting cancer and vice versa. The biological and biochemical pathways underlying this close relationship will be analyzed. In this new overlapping scenario, physical activity and exercise are proven protective behaviors against both cardiovascular diseases and cancer. Many observational studies link an increase in physical activity to a reduction in either the development or progression of cancer, as well as to a reduction in risk in cardiovascular diseases, a non-negligible cause of death for long-term cancer survivors. Exercise is an effective tool for improving cardio-respiratory fitness, quality of life, psychological wellbeing, reducing fatigue, anxiety and depression. Finally, it can counteract the toxic effects of cancer therapy. The protection obtained from physical activity and exercise will be discussed in the various stages of the cancer continuum, from diagnosis, to adjuvant therapy, and from the metastatic phase to long-term effects. Particular attention will be paid to the shelter against chemotherapy, radiotherapy, cardiovascular risk factors or new onset cardiovascular diseases. Cardio-Oncology Rehabilitation is an exercise-based multi-component intervention, starting from the model of Cardiac Rehabilitation, with few modifications, to improve care and the prognosis of a patient’s cancer. The network of professionals dedicated to Cardiac Rehabilitation is a ready-to-use resource, for implementing Cardio-Oncology Rehabilitation.

## 1. Introduction

Cancer and cardiovascular diseases (CVD) represent the leading causes of mortality and morbidity globally [1]. Even the recent epidemiological trend seems to overlap between cancer and CVD: in the U.S., mortality rates from cancer in middle-aged adults decreased by 19% between 1999 and 2017, and death rates from CVD decreased by 22% from 1999 to 2011 before increasing by 4% from 2011 to 2017 [2].

Furthermore, cancer and CVD share many risk factors, and justify a common prevention strategy, both in the population and at individual levels. A healthy lifestyle can reduce mortality from both cancer and CVD. In a Nurses’ health study (77,782 women between 34 and 59 years old, free of CVD and cancer in 1980, with a 24-year follow-up), mortality in relation to the presence of five lifestyle factors was analyzed: smoking, being overweight (body mass index (BMI) ≥ 25 Kg/m^2^), lack of physical activity, i.e., <30 min/day moderate to vigorous activity, low-quality diet and alcohol consumption [3]. The relative risks (for five vs. zero lifestyle risk factors) were 3.26 for cancer and 8.17 for CVD mortality. A total of 28% of deaths during follow-up could be attributed to smoking, and 55% to the combination of smoking, being overweight, lack of physical activity, and poor quality of diet.

Beyond sharing risk factors (diabetes, obesity, smoking), a common biology is emerging from the close relationship between CVD and cancer. Therefore, Cardiac Rehabilitation (CR) programs may be extended to cancer patients representing a valid therapeutic strategy aimed at improving the survival and quality of life (QoL) in patients with malignancy.

### Cancer and Cardiovascular Diseases: A Bidirectional Relationship

The state of chronic inflammation promoted by viral or bacterial infections, radiation, alcohol consumption, smoking or exposure to chemicals, but also due to dyslipidemia, obesity and diabetes, could be the basis of the atherosclerosis and carcinogenesis [4]. Physical activity reduces adipose tissue and decreases the production of sex and metabolic hormones, insulin, leptin, and inflammatory markers, several of which are potentially carcinogenic. Chronic exercise is associated with decreased levels of oxidative stress markers and increased enzymatic and non-enzymatic antioxidant capacity in the young, middle-aged, and elderly [5].

In a cohort study of 2839 patients with colon–rectal cancer, visceral adiposity and muscle radiodensity (i.e., muscle fat content) were associated with CV events [6]. Equally, but in the opposite direction, in postmenopausal women, higher levels of body fat are associated with an increased risk of breast cancer and altered circulating metabolic and inflammatory factors (higher levels of insulin, C-reactive protein, IL-6, leptin and triglycerides, and lower C-HDL and sex hormone-binding globulin) [7].

This common pathophysiology justifies, not only the increased CV risk of patients with cancer, but also the raised risk of cancer in patients with CVD. In 1081 subjects with heart failure (HF), after myocardial infarction at an average follow-up of five years, there was an increased risk of malignancy (Hazard Ratio (HR) = 2.16 adjusted for age, sex, and Charlson index) [8]. In the Mayo Clinic register [9], the HF/cancer relationship emerges, especially in recent years, characterized by a longer survival in conjunction with the greater efficacy of HF therapy. The higher cancer risk in subjects with a low ejection fraction suggests a relationship with impaired immunity and chronic inflammatory status, besides the shared risk and more intense medical surveillance. This association was confirmed in a Danish registry [10]. In a HF Japanese registry, the risk of developing cancer was present in those who had a baseline C-reactive protein (CRP) value > 2.0 mg/dL (HR 1.87) and remained high after one year [11].

The bidirectional relationship of cancer/CVD is also supported by epidemiological studies on atrial fibrillation, with important prognostic and therapeutic implications (evolution towards HF, increased thromboembolic and hemorrhagic risks) [12]. In the Outcomes Registry for Better Informed Treatment of Atrial Fibrillation (ORBIT-AF) registry [13], among 9749 patients, about 25% had a history of cancer (57% solid malignancy, 1.3% leukemia, 3.3% lymphoma, 40% other, and 2.2% metastatic cancer). Patients with history of malignancy were older, with a greater probability of having CVD, CV risk factors, and previous gastrointestinal bleeding. Once again, especially in women, a new onset of atrial fibrillation [13] is associated with an elevated risk of cancer (HR 3.54) in the first 3 months, which remains significant beyond 1 year (HR 1.42).

In some cases, a common genetic basis can be assumed: several forms of non-chromosomal congenital heart disease are at increased risk for hepatoblastoma and neuroblastoma [14]. The so-called clonal hematopoiesis of indeterminate potential (CHIP) is a mutation of hematopoietic stem cells in bone marrow, related to cancer formation (hematological malignancies) and also the development of CVD, especially HF [15].

Regarding CV drugs, there are concerns for thiazide diuretics and angiotensin-converting enzyme (ACE) inhibitors: the former could have a photosensitizing action, which increases the risk of skin tumors, the latter, through the rise of bradykinin, could increase the endothelial growth factor, thereby promoting angiogenesis [15]. However, there is no clear evidence so far of the carcinogenic effect of these widely used drugs. Other cardio-metabolic drugs have a neutral or protective action (aspirin, colchicine and metformin).

The efficacy of therapies and anticancer screening has increased, survival exposing patients to the risk of developing CVD, also as an effect of chemotherapy and radiotherapy. In the U.S., in 2016, more than 15.5 million patients with a history of cancer were estimated, and this number is expected to increase to 20.3 million in 2026 [16].

Left-sided chest radiotherapy and anthracyclines can cause heart damage during treatment, and even long after exposure. The CV risk profile can be compounded by any cancer therapy: irradiation of the skull or the neck may induce obesity secondary to hypopituitarism or hypothyroidism, prolonged hormone therapy in sensitive tumors can modify the metabolic profile of patients, some treatments can promote persistent endothelial dysfunction, determining the appearance of insulin resistance [17].

In more than 36,000 patients with cancer [18], the prevalence of CV risk factors was significantly higher than in subjects with no history of malignancy; eight-year survival was significantly worse among cancer survivors who developed CVD (60%), compared with cancer survivors without CVD (81%).

**Figure 1:**
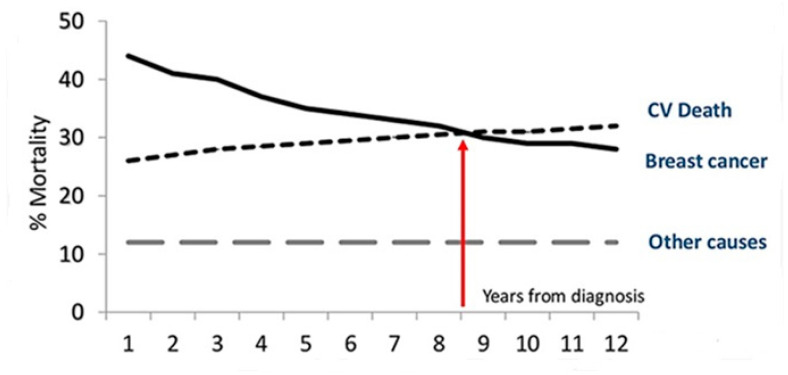
Proportional distribution of the main causes of death by time since the diagnosis of breast cancer.

The competitive risk of CV death is relevant and may exceed that of cancer progression or recurrence in postmenopausal women with breast cancer [19,20] and in men with prostate cancer [21]. The risk is conditioned by the presence of modifiable factors: cigarette smoking, obesity, metabolic syndrome and diabetes, high blood pressure, and a sedentary lifestyle. Of 66,000 women with breast cancer (mean age at diagnosis of 66 years), half survived after 12 years follow-up [22]; around the ninth year, the probability of CV death was equal to that of cancer, but, later, the main cause of death was CV (Figure 1). Therefore, reducing CVD risk should be a priority for the long-term care of patients after cancer diagnosis.

CV events are the leading cause of death among children and adolescent cancer survivors; they were significantly more likely than siblings to suffer from HF (HR 5.9, *p* < 0.001), acute myocardial infarction (AMI) (HR 5.0, *p* < 0.001), pericardial disease (HR = 6.3, *p* < 0.001), or valve abnormalities (HR = 4.8, *p* < 0.001). An anthracycline dose greater than 250 mg/m^2^ and a radiation dose higher than 1500 centigray significantly increased the risk of CVD [23]. Recently, reductions in exposure to cardiac radiation have been associated with a lower risk of coronary artery disease among adult cancer survivors [24].

Recently, the attention has focused on how the cardiac extracellular matrix in HF and tumor stroma can have a paracrine and endocrine crosstalk, mediated by fibroblasts (accentuated in diabetes and hypertension), able to stimulate the growth of latent cancer [15].

The bidirectional cancer/CVD relationship has recently been confirmed in a large observational study conducted in the US [25]. Among 3,234,256 cancer survivors (1973–2012), 38.0% died of cancer and 11.3% (365,689) of CVD. Analyses were adjusted by age, race, and sex. Most CV deaths (76.3% due to heart disease) occur in breast, prostate, or bladder cancer, with endometrial cancer presenting a very high risk of CV death the first year after diagnosis. CVD mortality, highest during the first year (especially in survivors diagnosed <35 years of age), remains high in the follow-up compared to the general population; cancer patients have an average two- to six-fold higher risk of CV mortality than the general population.

The early peak in the risk of CVD is followed by a chronic phase (survival period). There is a “sweet spot” where the risk of mortality remains stable for years; then, it seems to increase again as a consequence of the late toxicities of chemotherapy, or of the accelerated or natural progression of pre-existing diseases (both cancer and CVD). The chance to die from CVD overtakes the chance of cancer-related mortality when cancer survival time lasts longer. However, since both conditions share risk factors, biologically common substrates and harmful effects of some cancer therapy, it might be intuitive that cancer and CVD benefit from a healthy lifestyle.

The bidirectional relationship has the consequence that cardio-oncology must acquire a new look beyond the narrow confines of its birth, that is, the diagnosis and treatment of toxic effects induced by chemotherapy. Oncologists have to familiarize themselves with instruments with proven effectiveness in preventing and treating CVD: pharmacological therapies, such as beta-blockers and ACE-I (beyond protection against the cardiotoxic effect), drugs effective in CV prevention (statins, omega-3, antiplatelets anticoagulants), but also a healthy diet and lifestyle. Finally, it is mandatory to use a tool of proven efficacy, such as increasing physical activity through structured exercise programs, the core business of cardiac rehabilitation (CR).

The core component of CR and secondary prevention programs can be shared, being multi-factorial interventions, with cancer patients: health education, CV risk reduction, stress management, increased physical activity and exercise, healthy diet, optimization of drug therapy. The CR reduces mortality, morbidity and hospitalization, as well as improving exercise capacity, psychological wellbeing and QoL in patients with CVD [26,27,28,29,30,31,32,33,34,35,36,37,38,39,40,41,42,43,44,45,46,47].

It is time to extend the benefits of CR to cancer patients, at least in terms of improving functional capacity and QoL, and possibly becoming an integral part of cancer treatment.

## 2. Physical Activity and Exercise after the Diagnosis of Cancer

A meta-analysis on approximately 50,000 breast and colon cancer patients emphasizes how performing 150 min/week of moderate physical activity (PA) is associated with a reduction in all-cause mortality: −24% for breast and −28% for colon. In subjects who increased the level of PA after diagnosis, there was a reduction in mortality (RR = 0.62) compared to those who did not change it or who were already inactive before diagnosis [48]. Lahart et al. [49] reported similar data in a meta-analysis comprising 22 studies and 123,574 women with breast cancer: vigorous PA, as suggested by the guidelines (≥8 metabolic equivalents (MET)-hour/week), exerted a significant reduction in all-cause mortality (HR = 0.54) and in cancer–related death (HR = 0.67). Furthermore, PA before and after diagnosis is associated with fewer cancer-related events, such as recurrence (HR = 0.79) and tumor progression (HR = 0.72).

A recent meta-analysis evaluated the adherence rates to the aerobic physical activity guidelines among people with chronic conditions (CVD, diabetes and cancer) (*n* = 3721), as physical activity is an effective form of treatment and prevention of chronic disease [50]. Authors reported an average adherence rate of 77% (95% Confidence Internal (CI) = 0.68–0.84) of their prescribed physical activity treatment, regardless of underlying conditions. The pooled adherence rates for clinic-based and home-based programs did not differ (74% (95% CI, 0.65–0.82) and 80% (95% CI, 0.65–0.91), respectively). This data supports the hypothesis that people with chronic conditions are capable of sustaining aerobic physical activity for three or more months, as a form of treatment.

Moreover, exercise (E) programs, especially if supervised, improve QoL and physical function in cancer patients with different demographic and clinical characteristics (including C-type), during and after treatment [51]. An individual patient data meta-analysis confirms the data, underlining how the benefit is greater for subjects with high fatigue and low physical function [52]; moreover, in childhood cancer, E training improves functional mobility [53].

The evaluation of cardiopulmonary function may have prognostic relevance in cancer patients. The VO_2peak_ measurement in a group of approximately 250 women with malignancy (during adjuvant or metastasized therapy) shows an average value 27% lower than that of women of the same age and comparable to that of healthy women of approximately 20–30 years older, with lower values in women with metastases. The VO_2peak_ is a predictor of survival in metastatic disease [54].

Ventricular function is usually normal, indicating how other factors involved in oxygen transport (pulmonary, vascular, and skeletal muscle function) play an important role in determining the deterioration in the physical state. Exercise training can become a strategy to improve the prognosis in these patients. In 571 subjects with various types of cancer (breast, prostate and lymphoma), a program of supervised physical training with prescription of E three times/week, from moderate to intense (40–80% of peak frequency or VO_2peak_), lasting 15–45′, can induce a significant increase in maximum oxygen consumption (2.91 mL/kg/min) vs. a decline in the usual care group [55]. The training was similar to that usually practiced in CR, with the exception of the duration of the sessions and the higher incidence of adverse events.

Growing interest is focused on endothelial dysfunction since it shares common pathways in both cardiovascular and cancer diseases. In fact, playing a crucial role in neoangiogenesis and in regulating local blood flow, endothelial dysfunction and the disarray of newly formed vessels within the neoplastic mass massively influence the abnormal intra-tumoral perfusion, leading to local hypoxia, impaired permeability, extracellular matrix degradation and the efflux of cancer cells, determining a higher risk of metastatic diffusion [56]. A recent study showed that micro-vascular endothelial dysfunction, as defined by reactive hyperemia peripheral arterial tonometry index ≤ 2.0, was associated with a greater than two-fold increased risk of solid-tumor cancer [57]. Interestingly, in a small cohort of patients with breast cancer from the Mediterranean diet-based diet and androgens (DIANA)-5 Trial, a 12-month exercise training program improved both VO_2_peak (from 12.4 ± 2.9 to 14.3 ± 3.3 mL/kg/min, *p* < 0.001) and vascular endothelial function (Reactive Hyperemia Index from 2.1 ± 0.7 to 2.5 ± 0.8, *p* < 0.001) [58]. In addition, in women with breast cancer from the DIANA-5 study, an exercise-induced improvement in autonomic function (as measured by heart rate recovery), significantly correlated with the improvement in VO_2peak_ (r = 0.58, *p* = 0.002), was also reported [59]. There is a relationship between training intensity and the increase in functional capacity. A program of medium to high intensity endurance training (two supervised sessions of 30 min, Borg scale 12–16/20; and a third session of 30 min of home aerobic training) is more effective than a low-intensity home program (five sessions of 30 min, Borg 12–14/20) to antagonize the decrease in functional capacity during adjuvant therapy for breast cancer; both are better than usual care. At the 6-month follow-up, all three groups returned to their baseline level of fitness [60]. Moreover, medium to high intensity exercise increased muscle strength, reduced fatigue, and required minor adjustments in chemotherapy; both types of T were associated with less nausea, vomiting, and pain and a faster return to work, alongside an improvement in QoL.

Moreover, in operable breast cancer, stage IIb–IIIC, treated with neoadjuvant chemotherapy (doxorubicin, cyclophosphamide), participation in an aerobic training program (with resistance exercises three times a week for 12 weeks) can increase functional capacity (2.6 mL/Kg/min on average, +13.3%), which declined in the chemotherapy-only group [61]; furthermore, an aerobic training program can increase lean mass, muscle strength and fitness level, as well as reducing adiposity, blood pressure, blood lipids and improving glycemic control, effects particularly useful (reduced fatigue, vitality increased) in prostate cancer, especially in the case of androgen deprivation therapy [62,63]. A significant reduction in BMI and body fat, an increase in lean mass, respiratory fitness and reduced fatigue, were also proven in breast cancer and lymphoma, both in chemotherapy [64,65]. Therefore, exercise can mitigate the adverse metabolic effects of chemotherapy and radiotherapy.

A training program, coordinated by a nurse, in the form of tele-rehabilitation [66] can have a positive effect in patients with solid or hematological cancer, even in late stage (IIIC or IV). There was an improvement in fitness and QoL, and a reduction in the length of hospitalization and the use of post-hospital care.

The exercise proved to be useful and feasible even in patients with advanced cancer and reduced ventricular function. In 65 women [67] with metastatic breast cancer (stage IV, 57% in CT and 40% with ≥2 previous lines of therapy and 34% with CV comorbidity), the group treated with an anaerobic training program (12 weeks, three sessions a week of 15–45 min at 55–80% of VO_2peak_) showed a significant increase in functional capacity; however, exercise was tolerated only in 63% of the participants. In breast cancer, where a reduction in anthracycline-induced ejection fraction (EF) was observed in both the supervised training group and normal care; exercise, however, attenuated the decrease in VO_2peak_ [68] and also the cardiotoxicity of anthracycline, as evaluated by global longitudinal strain [69].

Exercise should be considered as a drug and, consequently, adherence to the program is crucial. In the Heart Failure: A Controlled Trial Investigating Outcomes of Exercise Training (HF-ACTION) study in the subgroup of patients with cancer and heart failure, aerobic training improved outcomes (all-cause mortality and hospitalization) only in adherent (≥90 min E per week) compared to non-adherent patients [70]. Predictive factor adherence is brief chemotherapy, lack of endocrine symptoms, lower functional limitation, higher VO2peak, earlier stage of the disease and, finally, an aerobic training protocol [71].

Exercise during chemotherapy is also a strategy to minimize long-term treatment-related side effects. In breast or colon cancer patients, the higher the level of exercise (moderate-to-vigorous), the lower the fatigue at 4 years post-baseline [72]. In a large prospective observational study involving 992 patients (stage III colon cancer underwent adjuvant CT) followed for a median of 7 years, a healthy body weight (BMI between 25 and 29), a diet rich in vegetables, fruits, and whole grains, and a moderate level of PA (8.75 MET-hour/week, approximately 150′ of brisk walking) were conditions associated with a 42% reduction in the risk of death [73]. The number of patients who would need to adopt a lifestyle consistent with guidelines to prevent one death at 5 years (NNT) was 12.

## 3. Protective Effect of Exercise on Cancer Progression

Several studies have proposed the possible biological mechanisms underlying the protective effect of exercise on cancer progression. An anti-inflammatory effect of exercise has been suggested by the reduction in the circulating levels of the nuclear protein HMGB1 (high-mobility group Box-1) [74], which binds to DNA by promoting the transcription of specific proteins; HMGB1 also functions as an extracellular signaling molecule during inflammation, cell differentiation, cell migration, and tumor metastasis. Once again, reduced HMGB1 levels are proportional to exercise adherence [74].

In breast cancer patients undergoing adjuvant chemotherapy, exercise has been demonstrated as promoting a reduction in markers of inflammation. A structured training program reduces protein oxidation and the level of 8-hydroxyguanosine, a biomarker for oxidative damage [75]. The increase in antioxidant capacity correlates with a reduction in cancer-associated fatigue [76]. It is not yet clear whether the systemic antioxidant action of the exercise is a result of a change in the microenvironment of the tumor, or an alteration in systemic levels of oxidative stress [77].

Moreover, exercise increases vascular endothelial growth factor levels [78,79] and vascular density, while promoting better tumor perfusion [80]. The increment of shear stress induced by exercise has a positive vascular remodeling effect too [81]; the final result is a reduction in cancer tissue hypoxia.

Hypoxia is a protective factor for neoplastic growth, since it makes the tumor less sensitive to chemotherapy and to radiotherapy, also reducing chemotherapy-induced apoptosis [77]. The consequence is an increase in the effectiveness of chemotherapy combined with exercise, as demonstrated in the experimental model for cyclophosphamide, gemcitabine and doxorubicin [78,81]. The exercise-induced improvement in endothelial function (evaluated with flow mediated dilation of the brachial artery) is likely due to increase NO synthesis and the mobilization of endothelial progenitor cells. The end result is a better tumor vasculature, with a higher sensitivity to chemotherapy, which could result in a favorable alteration in the gene expression, with a change towards a less aggressive cancer phenotype [82,83]. A trial is currently underway to evaluate the effects of a high-intensity interval training program associated with hyperoxia or hypoxia in patients during chemotherapy [84]. Therefore, synergistic action with chemotherapy can be hypothesized.

The better vascularization also reduces interstitial pressure and increases vascular permeability [85], favoring an easier, interleukin-6 mediated, infiltration of T lymphocytes in the tumor by the up-regulation of endothelial adhesion molecules. Furthermore, an interval training protocol in women with breast cancer, stage I-III, undergoing chemotherapy was able to induce a significant increase in natural killer cells immediately after exercise; the raised cardiac output is likely to act synergistically with the increased adrenergic tone by altering the endothelial adhesion capacity of lymphocytes, thereby recruiting natural killer cells from the periphery and deposits [86]. A systematic review [87] confirms the immuno-stimulatory function of exercise by an increase in the number and cytotoxicity of monocytes, natural killer cells and cytokines.

Finally, exercise can change the metabolism of the tumor. Neoplastic cells preferentially use glycolysis compared to aerobic metabolism for hypoxia and the high cell growth associated with oxidative stress [88]. The training can reduce glucose consumption and lactate production and increase fatty acids oxidation [77]. The final result is a shift towards aerobic metabolism with a greater sensitivity to cancer therapy [89]; besides the reduction in lactate, raising the pH could improve the response to immunotherapy and increase the number natural killer cells [77,88].

## 4. Protective Effect of Exercise on Cardiovascular Risk Factors and Cardiovascular Events in Cancer Patients

Pre-cancer diagnosis levels of physical activity also reduce subsequent CV events [90]. A cohort of 4015 women with primary breast cancer were enrolled in the Women’s Health Initiative and completed a questionnaire on leisure time physical activity assessed by METs/h/week. The data was collected prospectively, and age and multivariable adjusted. Median follow-up was 12.7 years and 8.2 years for CV mortality and CV events. The incidence of CVD decreased with the increase in total METs categories (*p* = 0.016). Compared to <2.5 METs/h/week, the HR was 0.80 for 2.5 to <8.6 METs/h/week; HR 0.9 for 8.6 at <18 METs/h/week and HR 0.63 for >18 METs/h/week. Clearly, patients performing higher levels of pre-diagnosis exercise will probably have a more favorable CV risk profile; however, data underline the beneficial effect on CV risks of the physical activity. Moreover, the study shows that only a third of patients meet the recommendations of 150 min of moderate to vigorous PA per week, and very few perform 18 METs/h/week.

In patients with Hodgkin lymphoma, physical activity was assessed with a questionnaire [91]. The primary end point was the incidence of any CV event with a median follow-up of 11.9 years. Adherence to the guidelines, for vigorous intensity exercise (i.e., ≥9 h METs/week) was associated with a 51% reduction in the risk of any CV event compared to non-adherence to the guidelines (*p* = 0.002). Exercise was associated with a lower risk of CV events in a dose-dependent manner, regardless of CV risk profile and treatment.

Similar findings were observed in a study carried out in 2973 women with non-metastatic breast cancer, (average age 57 years), and a follow-up of more than 8 years, where patients following the guidelines (≥9 METs/h/week) translated into a 23% reduction in the risk of CV events [92]. The protective effect is independent of other factors such as age, menopause, antineoplastic therapy and, above all, the previous CV risk.

Metabolic syndrome, defined as a cluster of CV risk factors in cancer survivors, could be the late consequence of hormone therapy, chemotherapy, radiotherapy and surgery [17]. Exercise is one of the intervention strategies. Patients initiating radiotherapy with or without androgen deprivation therapy were randomly assigned to usual care, resistance, or aerobic exercise for 24 weeks. Resistance training in addition to improving fatigue, functional capacity, QoL, and strength in the extremities reduced serum triglycerides, and decreased body fat [62,63].

In a Canadian study,63 survivors of testicular cancer with history of CVD [93] were enrolled. The exercise program lasted for 12 weeks, three times a week, and was aerobic interval training, with periods of 4 min up to 95% of the VO_2peak_, followed by recovery intervals below the ventilatory threshold. In addition to increasing cardio-respiratory fitness, and also improving some surrogate parameters of CV risk, the program succeeded in reducing C-LDL, CRP, arterial stiffness, intimal thickness and increasing parasympathetic tone. The result was a 20% reduction in CV risk factors.

Exercise is an effective tool to oppose CV risk factors and CVD and also alterations induced by chemotherapy and radiotherapy, which are a model of accelerated atherosclerosis.

Recently, a prospective randomized single-center clinical study involving 100 women with breast cancer (stage I–III), who were sedentary, overweight or obese (BMI ≥ 25.0 or body fat ≥ 30%) with chemotherapy completed within the previous 6 months was published [94]. CV risk was assessed with the Framingham Risk Score for each participant, using a preset point for each of the 6 Framingham Risk Score categories: age, systolic blood pressure, C-HDL, C-LDL, diabetes and smoking. The exercise group underwent supervised aerobic and resistance training sessions three times/week for 16 weeks, reducing the predicted 10-year Framingham Risk Score by 11%.

The assessment of CV risk factors is mandatory, not only at the moment of cancer diagnosis, but also later. In breast cancer patients, treated with anthracyclines and/or trastuzumab, the women who developed late cardiac toxicity showed CV risk factors at the time of toxicity that were not reported at the diagnosis, which is why their control should be part of the survivorship program [95].

In conclusion, the effects of exercise treatment in oncology can be defined as bimodal. In the early stages of the disease, they enable an increase functional capacity, reduce disabilities and improve QoL and psychological wellbeing; moreover, they can antagonize the negative effects of chemotherapy and radiotherapy (and perhaps improve their effectiveness). Later, exercise, in a dose-dependent manner, can prevent morbidity and mortality and cancer recurrence; moreover, it clearly reduces CV events, which, at a distance from diagnosis, especially for some types of cancer, represent the main cause of death.

## 5. Exercise-Induced Protective Effects on Anthracycline Damage

Exercise increases the availability of calcium transporters in the sarcoplasmic reticulum, improves inotropism, and has positive effects on endothelial and mitochondrial function in animal models [96]. Of these beneficial effects, only the reduction in the natriuretic peptide has been confirmed in humans, after an endurance training program in women with breast cancer [58,59,97].

Exercise increases survival in anthracycline-treated animals. The main protective mechanism of aerobic training is mediated by protection from reactive oxygen species (ROS) damage, both through reduced production of superoxide anions, and through increased expression of antioxidant enzymes (catalase, glutathione peroxidase, and manganese superoxide dismutase). The final result is the prevention of the denaturation of intracellular proteins, and the acceleration of their repair [98]. Exercise not only prevents the doxorubicin-induced increase in apoptosis mediators, but a short training program (21 days) reduces the apoptotic index by about five times [99,100].

In addition to cell death reduction, exercise can significantly increase the proliferation of cardiac progenitor cells. The training increases GATA 4, a protein involved in myocyte differentiation and stimulated by α-1 adrenergic activity, which induces the negative regulation of inhibitory factors and an increase in those involved in the proliferation and hypertrophy of myocardial cells [101]. Moreover, it seems to reduce the calcium overload, optimizing the function of the sarcoplasmic reticulum, stimulating the deterioration in the calpain, a protein that promotes the degradation of proteins binding calcium [102].

An unproven mechanism of the exercise-induced prevention of anthracycline damage is the increased activity of adenosine monophosphate (AMP)-activated protein kinase [103], which has a crucial role in the homeostasis of myocardial energy, through of the activation of catabolic pathways for the production of ATP (such as the oxidation of fatty acids).

## 6. Protective Effect of Exercise on Molecularly Targeted Therapy Damage

Tyrosine kinase receptor inhibitors represent the most important class among molecularly targeted therapies. The receptors found in the cell membrane are involved, not only in the regulation of many cellular processes, but also in malignant transformation and tumor proliferation if their activity is amplified by a genetic abnormality or mutation [104]. The excessive expression of the HER2 receptor (also known as ErbB2) is present in approximately 20% of breast cancer, in 10% of lung adenocarcinomas, and in 5% of gastric cancer [105].

Receptor blocking through complex metabolic pathways increases the expression of CCAAT-enhancer-binding proteins (C/EBPs) a family of transcription factors involved in inhibiting cell proliferation, blocking the GATA synthesis. The result is a deterioration in the ejection fraction, leading to HF. In animal models, exercise induces the increased expression of neuregoline [106], an endothelial protein that binds to the ERb receptor with a final cardio protective effect [107]. It also reduces angiotensin, which, like adrenergic agonists, inhibits neuregoline [106].

Exercise increases phosphoinositide-3-kinase (PI3K), which has a fundamental role in inducing the hypertrophic response [107]. The rise in PI3K increases the activity of AKT protein kinase B (PKB), a cytosolic protein that activates biochemical pathways, with the end result being cell growth and a resistance to apoptosis. Exercise increases adenosine monophosphate kinase (AMPK), which stimulates fatty acid oxidation, glucose uptake and, in turn, activates the protein peroxisome proliferator-activated receptor gamma coactivator 1-alpha (PGC-1α), which activates mitochondrial function. The aforementioned increase in the activity of GATA4, induced by exercise, modulating the degradation of proteins and elevating their synthesis, promotes the hypertrophic response.

These protective mechanisms are speculative and are based on biochemical pathways demonstrated only in the experimental model. To our knowledge there is only one clinical study in women with breast cancer, treated with trastuzumab, in which 16-week exercise training did not reduce ventricular dilation, probably due to a low adherence to the protocol [108].

Drugs with antiangiogenic action block vascular endothelial growth factor (VEGF). Its stimulation is associated with the activation of the NO pathway and the mobilization of myocardial progenitor cells. Therefore, antagonizing VEGF led to the blocking of the migration, proliferation, growth and formation of new vessels and a new capillary network, which is essential for neoplastic diffusion [109]. At a CV level, it also reduces availability of NO, induces vasoconstriction, increases peripheral resistance, and blood pressure. In addition, it also counteracts myocardial cell differentiation by limiting progenitor cells [105].

E increases PGC-1α protein transcription, which, in turn, raises the expression of VEGF. In addition, PGC-1α increments the number of mitochondria and the fibers of oxidative metabolism (slow contraction fibers type I) in the skeletal muscle, increasing resistance to fatigue [110]. Finally, exercise can increase cardiac progenitor cell production and mobilization through an NO-mediated increase in VEGF, the release of IL-6, and the increase in signal transducer and activator of transcription 3 (STAT3) activated by cytokine and growth factors.

The final result is the stimulation of hypertrophy and cell differentiation with protection against ischemia and pharmacological damage [111].

## 7. Exercise-Induced Protective Effect on Radiotherapy Damage

Radiotherapy (RT) exerts negative effects on CVD, accelerating coronary artery disease, and determining conduction system damage, constrictive pericarditis, and vascular and valvular damage. These might explain the early onset of CV events, and the consequent increase in mortality and morbidity [23,24].

An additional negative effect of radiotherapy has also been described: autonomic dysfunction (AD) identifies the loss of the normal autonomic regulation of the CV system, associated with the excessive activation of the sympathetic nervous system, not counteracted by adequate parasympathetic activity [112]. Increased heart rate is the main manifestation, but also faster atrio-ventricular node conduction, contractility, and oxygen consumption. Besides, is well known that a high heart rate per se is able to promote atherosclerosis.

The basal heart rate of women treated with adjuvant chemotherapy for breast cancer is, on average, seven to 16 beats faster compared to age-matched controls [113]. A sample of 4876 women with breast cancer (stage I–III) were followed for an average of 5 years [114]; an increase of 10 beats/min entails an incremental risk of all-cause (15%), cancer specific mortality (22%) and cancer recurrence (6%). In patients with colon cancer, a heart rate > 80/min, after adjustment for various risk factors, is associated with an increased risk of recurrence (HR = 6.18) compared to heart rate < 66/min. This association is stronger in sedentary people with a higher percentage of body fat [115].

Another tool for to valuating the AD is heart rate recovery, which is the reduction in heart rate after 1 min following the peak of exercise stress testing (if <12 beats per minute is an index of reduced parasympathetic tone reactivation, a surrogate marker of autonomic dysfunction).

The exposure of the neck to irradiation and damage to the vagus nerve and carotid sinus during radiotherapy, strategies more widely used in the past, explain the temporal trend of autonomic dysfunction, with a higher prevalence in subjects treated in the past, especially for Hodgkin lymphoma. In 263 subjects [112] with previous radiotherapy for Hodgkin undergoing exercise testing, the occurrence of radiotherapy, after correction for age, therapy, sex, CV risk factors, and stress test indications, correlates with an increased risk of autonomic dysfunction, with an odds ratio (OR) of 3.96 for elevated baseline heart rate, and OR 5.32 for an abnormal heart rate recovery. Autonomic dysfunction is also related to a reduced functional capacity. Abnormal heart rate recovery, but not a high heart rate at rest, increases all-cause mortality (OR=4.60).

In addition to radiotherapy, chemotherapy can also induce autonomic dysfunction: anthracyclines, trastuzumab, taxanes and cyclophosphamide are able to induce an increase in basal heart rate. The raised level of circulating catecholamine levels was demonstrated before a detectable reduction of EF [116]; obviously, the onset of overt HF can accentuate it. Additional conditions modulating the autonomic dysfunction are psychosocial stress, alterations in sleep patterns, weight gain (sarcopenic obesity) and low fitness. This multi-factorial origin justifies a disproportionate autonomic dysfunction, compared to the lower reduction in the ejection fraction, comparing cancer patients with those with HF [117].

In 1070 consecutive patients undergoing a 12-week supervised phase II CR program, an abnormal heart rate recovery led to a risk of all-cause death (HR 2.15), but in those in which it was normalized, the mortality was comparable to subjects with absent autonomic dysfunction [118]. Similar data are lacking in the field of cardio-oncology. However, a moderate 16-week Nordic Walking exercise program can improve the sympatho-vagal balance in patients with malignancy [119]. In women with operable breast cancer receiving neo-adjuvant chemotherapy, an aerobic exercise program has a mitigating effect on the basal heart rate [58,59]. The autonomic dysfunction is able to predict a reduction in functional capacity, mortality and cancer recurrence. All therapeutic tools, pharmacological and not, must be implemented to reduce it. Among these, exercise appears to be effective and promising, but, unfortunately, is underused.

## 8. Cardiac Rehabilitation as a Model of Cardio-Oncology Rehabilitation (CORE)

The beneficial effect of training, especially supervised, should extend from the field of CR to that of patients with cancer [120]. The positive effects on QoL, functional capacity, psychological state and mood were demonstrated; however, above all, a positive impact on morbidity, mortality and cancer recurrence is an intriguing hypothesis. We are also beginning to understand the biochemical pathways underlying the protective action against chemotherapy and radiotherapy. Lastly, but by no means unimportantly, exercise has an important protective effect on CVD, which represents a major cause of death in cancer survivors.

Therefore, it seems fully justified to introduce the term Cardio-Oncology Rehabilitation (CORE), since, surely, a rehabilitation program based on supervised exercise could be useful in cancer patients also undergoing on chemotherapy and radiotherapy, especially if they have ventricular dysfunction. In addition, training may also be useful in some types of cancer (such as breast, colon, lung, prostate, lymphomas and hematological diseases) where the associated CV risk it is not negligible, to fully exploit the therapeutic action of CR. Finally, increasing cardio-respiratory fitness could be a tool to achieve benefits in relation to symptoms, mortality and recurrence in all types of cancer.

Rehabilitation in oncology still means, for many health professionals, a way to minimize the effects of surgery (retractions, lymphedema, correct posture) and improve autonomy in everyday life.

It is time to extend the benefits of CR to cancer patients. Local experiences have existed for some time, but, recently, the American Heart Association, with the endorsement of the American Cancer Society, has published an important statement on CORE [121].

The availability of a network of professionals dedicated to CR is a ready-to-use resource. CR units are distributed throughout the country, albeit non-uniformly, and represent a place for CORE in the treatment of cancer.

**Figure 2:**
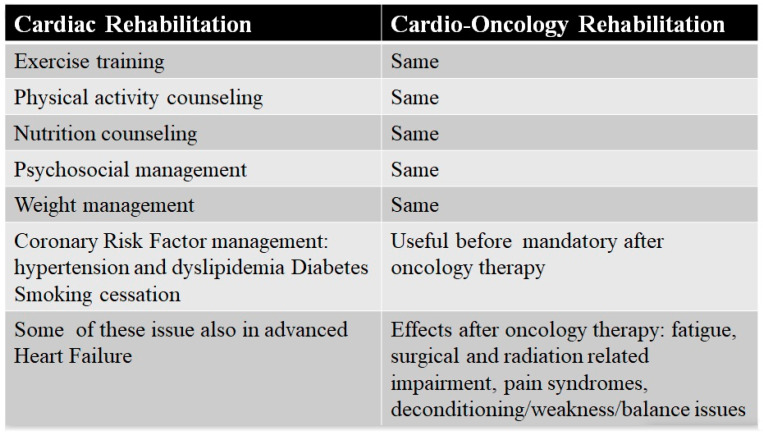
Core components of cardiac and Cardio-Oncology Rehabilitation programs.

Dittus et al. [122] were the first to propose the CR model, with few changes, also for oncology. The overlap between the core components of the CR and the CORE is surprising (Figure 2). Exercise programs (aerobic or resistance) to increase functional capacity are the same, as are psychological supports to reduce stress and quit smoking and advise patients to avoid unhealthy food and to check dietary changes. A dietitian support for evaluating nutritional status is essential, especially when sarcopenia and cachexia are concomitant conditions, or in the case of cancer-specific recommendations. A lipid control and weight management program with the evaluation of lean and fat mass is essential, especially when cancer is associated with metabolic syndrome, which is induced by chemotherapy [17].

There are numerous reports on the correlation between the inflammatory power of the diet and tumors [123,124,125,126]. Therefore, chronic inflammation is the key factor to be countered to reduce the incidence of chronic diseases including CV diseases and cancer. Meat-poor food patterns such as the Mediterranean diet have a low inflammatory index. A “non-Western” low-calorie food pattern, Mediterranean or lacto-ovo vegetarian diet reduces, within 3 months, oxidative stress and inflammatory cytokines in a similar way, except for interleukin-17, which improved more only with the Mediterranean diet [127]. In the Prevención con Dieta Mediterránea (PREDIMED) study, a reduction in CV events and a lower incidence of breast cancer in diabetic or high-risk CV women was observed when randomized with the Mediterranean diet, especially if accompanied by the regular use of extra virgin olive oil [128]. The beneficial effects of the Mediterranean diet are generally attributed to the action of nutrients within food, but is also able to influence the system of endogenous defenses through two mechanisms which involve areas of intense research: the intestinal flora (microbiota) and some compounds taken with the diet (i.e., bio-flavonoids) [129].

This is why the American Cancer Society Guidelines for cancer survivors recommend a dietary plan rich in vegetables, fruit, and whole grains, in order to limit the consumption of processed meat, red meat and alcohol (no more than one drink per day for women or two drinks per day for men) [130]. Unfortunately, the adherence to recommendations is globally low and inadequate among cancer patients, especially in long-term survivors: it is higher for non-smoking and low alcohol intake advice, but lowest for fiber intake [131]. Therefore, the effort to stimulate behavioral and dietary changes must be mandatory in the care of cancer survivors, but, above all, CORE is the right phase of the cancer continuum in which to apply them.

The combined intervention of nutrition therapy and exercise has been shown to have greater efficacy in relation to cancer fatigue in men with prostate cancer [132]. In addition, it has proven effective in reducing morbidity and mortality. In the Life After Cancer Epidemiology study, involving women diagnosed with early-stage breast cancer, a diet that followed the American Cancer Society guidelines was associated with a significant decrease in the risk of all-cause mortality, but not a reduced risk of specific breast cancer mortality or recurrences [133]. This might depend on the specificity of the cancer. In the Women’s Intervention Nutrition Study on 2437 women between the ages of 48 and 79 years with early-stage breast cancer, with a follow-up of 20 years, the benefit of a low fat dietary intake was shown only in women with negative estrogen receptor cancer [134]. In the Iowa Women’s Health Study of 938 breast cancer survivors, an intervention comprising exercise, weight loss and dietary advice reduced all-cause (HR 0.67), and CV mortality by 40% [135].

A multimodal intervention in cancer survivors (exercise, diet and other components of CR), may be critical to reduce mortality, morbidity and to reduce CV risk. In a subset of patients who are overweight or obese, the magnitude of the effect seems greater. CORE is the right time and the right way to apply these interventions.

Cancer treatment negatively impacts the cardiovascular system by determining hormone deficiencies, changes in insulin sensitivity, lipid metabolism, and inflammatory status. These changes can worsen the cardio-metabolic risk by promoting the development of metabolic syndrome [17,136]. There is a “common soil” between cancer, CVD and metabolic syndrome: obesity, insulin resistance, chronic subclinical inflammation dyslipidemia and hypertension. An unhealthy diet is the link that often binds these conditions, to which specific mechanisms are added for each type of cancer [137]. These are often in crosstalk.

Firstly, patients’ hormonal disorders, secondary to local therapies (e.g., orchiectomy or thyroid irradiation) or general therapies such as chemotherapy or hormone therapy, increase the risk of metabolic syndrome. Some examples are the blocking of the hypothalamic–pituitary axis caused by radiotherapy in survivors of childhood brain tumors [138], hypothyroidism induced by neck irradiation in Hodgkin lymphoma, the sarcopenic obesity induced by low testosterone levels in prostate cancer patients treated with deprivation androgen therapy and in breast cancer survivors in anti-estrogen therapy [136].

Hormonal disorders are also amplified by chemotherapy, possibly due to a greater sensitivity of the endocrine cells to the toxic action of certain drugs [17]; moreover, the chemotherapy can interact with the receptors and induce the dysfunction of the hormonal axis [139]. In patients with seminoma, the hypogonadism is more frequent in subjects treated with orchiectomy associated with cisplatin [140] or in survivors of acute childhood lymphatic leukemia [141]. Chemotherapy can induce insulin resistance and metabolic syndrome, with direct action even in the long-term period and through multiple potential mechanisms, especially if it is carried out at the pediatric age, a crucial period of development of the organs involved in metabolic homeostasis, such as the bone marrow, the muscles, the liver, the gastro-intestinal system, the adipose tissue and the endothelium [142].

Alkylating agents, anthracyclines, platinum derivatives and bleomycin produce reactive oxygen species, which mediate the anticancer effects and also lead to mitochondrial dysfunction in the healthy cells of numerous organs [136]. Other agents such as capecitabine promote the hepatic steatosis associated with decreased insulin sensitivity [143]. The vinca alkaloids and taxanes interfere with the microtubule system by blocking cellular replication, but also disrupt the important metabolic functions involved in glucose metabolism and muscle metabolism, liver production of lipoproteins, caloric intake and the composition of the microbiota, slowing down bowel motility. Endothelial dysfunction, induced by many chemotherapy agents, particularly by VEGF inhibitors, causes reduced vasodilation, inflammation, apoptosis and the impaired synthesis of nitric oxide [136]. The cytotoxic damage, apoptosis and anemia induced by chemotherapy lead to the release of cytokines and the activation of macrophages, alongside the development of obesity, insulin resistance, and dyslipidemia prodromal to metabolic syndrome [144].

There is also an “indirect” mechanism that can favor the development of metabolic syndrome. Muscle atrophy is frequent during chemotherapy and is associated with reduced insulin-mediated uptake [142]; maintaining an adequate level of physical activity can be a valuable tool to counteract the reduction in muscle mass.

A structured exercise program can reduce the prevalence of metabolic syndrome. In adults aged 50 to 65 years, increased moderate-to-vigorous activity is associated with its reduction (OR 0.33) [145]. An inverse relationship between fitness and metabolic syndrome probably also exists in cancer survivors: when BMI and waist circumference increase, fitness decreases, at least in survivors of breast and endometrial cancer [136].

Some types of cancer such as breast, colon, prostate and lymphomas seem to benefit more from CORE. In any case, the competence of the cardiologist and oncologist are often essential for the referral of the patient to CORE on the basis of age, comorbidities, CV risk factors and medical history, especially with regard to the time of exposure to cancer therapy.

However, the American Heart Association statement [121] underscores the fact that CORE is mandatory in the presence of high-dose chemotherapy and radiotherapy (high-dose anthracycline or radiotherapy ≥ 30 Gy), but also in the case of lower doses, if associated with ≥2 risk factors, older age, valvular disease, myocardial infarction or reduced ejection fraction. Finally, chemotherapy with low doses of anthracycline associated with trastuzumab is also a criterion for CORE.

Regarding CORE timing, there are two possibilities: CORE can be implemented at the time of diagnosis and during therapy or based on exposure to therapy and the onset of symptoms [121].

Although the first approach is pathophysiologic, exploiting the synergistic action of exercise with chemotherapy, deconditioning, fatigue, depression and side effects can make adherence to the CORE program more difficult.

The CORE team (doctor, physiotherapist, nurse) must have the skills not only to deliver a tailored exercise program, but also to evaluate the risks associated with the type of cancer and its therapy (HF and hypertension induced by chemotherapy, myocarditis and pericardial effusion from immunotherapy [146] or radiotherapy). In close collaboration with the oncologist, the patient’s CV risk profile must be evaluated, both basal and after cancer therapy, as well as the anatomical and functional limitations derived from cancer (deconditioning, fatigue, lymphedema, surgical direct injury).

A careful analysis of the patient’s unhealthy habits (smoking, obesity, level of physical activity), as well as any non-cancer-related therapy will be mandatory. The blood pressure should be measured twice in both arms, considering particular situations such as lymphedema or the subclavian steal in patients treated with mediastinal or neck irradiation [121]. It is essential to rule out orthostatic hypotension, which could increase the risk of traumatic injury during the training. The definition of good blood pressure control must take into account previous drug therapy, CV risk level, the hypertensive effect of chemotherapy and the beneficial hypotensive effect of exercise.

The control of diabetes mellitus must be one of the main objectives, as well as determining the presence of complications, stimulating self-management and underlining how diet and exercise can improve its control, especially in the case of chemotherapy (corticosteroids, androgen deprivation therapy), which can worsen glycemic control [121]. All strategies, not only to quit smoking, but also to avoid exposure to environmental smoke, must be adopted.

During CORE, fasting total cholesterol, C-HDL, C-LDL and triglyceride levels should be determined in subjects with CV disease history, with the goal of a C-LDL level below 70 mg/dL [121]. It is reasonable to also obtain this information in patients with ≥2 CV risk factors or metabolic syndrome. Although CVD risk score is useful [94], cancer-related factors, such as chest and mediastinal irradiation, must be considered to customize the definition of individual CV risk [23,24].

The definition of the body composition to determine fat and lean body mass and weight loss is crucial. Psychological intervention is a cornerstone for supporting cancer patients, increasing adherence to therapy QoL and reducing anxiety and depression.

Only starting from this complete basal analysis will the CORE team be able to set up a tailored aerobic and resistance training program to achieve the objectives. The functional assessment can begin even after a 6-min walk test, but periodic re-evaluations will be necessary to evaluate the achievement of objectives and to reshape the workload in the gym, based on the improvement in fitness.

A stress test or, better still, a cardiopulmonary exercise test, is essential as soon as possible; the target for moderated aerobic training is 70–85% of the maximum heart rate, with perceived exertion on the Borg scale of 10–12 (ranging from zero to 20). The program should be customized, including resistance exercises, on the level of conditioning. Resistance exercises are based on repetitions, starting at 30% of maximum load up to 60–70% for eight to 10 repetitions, until muscle fatigue of the major muscle groups (usually lower limbs) [121]. Usually, the program starts supervised and then can continue at home; telemedicine tools (tele-surveillance and tele-rehabilitation) could be useful.

After cancer therapy, poor conditioning, fatigue, and mobility deficits, sometimes due to surgery, may require specific interventions, as in the case of lymphedema in women treated with mastectomy. However, breast cancer survivors may find aerobic exercise more useful than home lymphedema care in order to improve symptoms [147].

Moreover, given the relevance of CVD as a comorbidity, but also as a risk factor for the toxicity of chemotherapy and, in long-term survivors, as a cause of death, it is mandatory to provide educational sessions on the management of CV risk factors, including stress reduction and smoking cessation. Oncologists will have to participate in the educational intervention, not only to explain the effectiveness of the therapy, including the usefulness of the training, but also the negative effects (cardiovascular and otherwise) of cancer therapies.

Therefore, through CORE, the old peripheral rehabilitation intervention must be integrated with central intervention (CR-derived intervention) based on physical training in order to achieve all the benefits of CR (cardio-respiratory fitness, QoL, risk factor control, etc.), and possibly also a reduction in total CV, oncology-related mortality and cancer recurrence.

The widespread adoption of the CORE model can also stimulate research in order to fill some knowledge gaps, including being able to identify [121] (1) which patients can benefit most from the CORE and the best time to adopt it, (2) to analyze the impact not only on CV outcome, but also on cancer-specific morbidity and mortality, (3) to define the best method of training (supervised, home, tele-rehabilitation or hybrid) with a related cost-effectiveness analysis and, (4) finally, to develop a model of automatic referral, especially for the categories of patients at highest risk.

## 9. Peculiarities of Cardio-Oncology Rehabilitation vs. Cardiac Rehabilitation

The heterogeneity of the response to physical training [120] is a fundamental element in CORE: for the same protocol, the response in terms of improving cardio-respiratory fitness can be located on a spectrum, ranging from neutral to positive and negative. Moving from a “one-size-fits-all” to a custom approach could reduce this heterogeneity.

The first element of personalization is individualization [148]. For the definition of the load, an exercise test should be carried out, avoiding using the age-predicted heart rate, for the risk of overtraining—the functional capacity of a patient with breast cancer is comparable to that of a healthy subject 20–30 years older [54]. The use of lactate sampling or anaerobic ventilatory thresholds [148] seems to allow better customization of the workload compared to VO_2peak_.

Cancer is a biologically heterogeneous disease. Physical activity reduces mortality by 50% in breast cancer with estrogen receptors, while it has no effect in the case of tumors without receptors [149]. Similar data have been described in patients with colon cancer, with a better cancer-specific survival only among nuclear β-catenin (CTNNB1)-negative subjects [150]. Even in women with breast cancer with a BRCA gene mutation, physical activity does not appear to play a protective role [151]. Usually, patients of a healthy weight responded better to the higher-dose E interventions than overweight/obese patients during chemotherapy for breast cancer [152]. Therefore, interactions between cancer and host factors can influence tumor aggressiveness; furthermore, factors related to the host, including genetics, immune competition, inflammatory status and intestinal microbiota, could play a role. Proper tumor and patient evaluation would maximize the effect of exercise.

The second factor to consider is the specificity of training. If reduced exercise tolerance is related to a cardiac output deficit, aerobic endurance is the most appropriate training. In the case of a peripheral limitation (reduced capillary density and reduced capacity to extract oxygen from the skeletal muscle), resistance training, coupled with aerobic training, is the most appropriate method. If the rapid recovery of fitness is required, interval training is the protocol of choice, especially if diabetes, dyslipidemia, and hypertension coexist with ventricular concentric remodeling.

Balance exercise can be useful if the patient has equilibrium problems, which are easily recognizable by placing the subject on one limb, as in chemotherapy-induced neuropathy [153]. In a systematic review, including 18 trials on breast cancer, exercise intervention improved social and physical functioning, but only medium-length sessions (>45 to ≤60 min; *p* = 0.03) and longer sessions (>60 to 90 min; *p* = 0.005) considerably improved QoL [154].

The third factor is the progression of the workload. In observational studies, the protective effect increases with workload [49,73], and there is a threshold effect, below which the effect of physical activity on mortality and cancer recurrence seems to be reduced. Therefore, in intervention training studies, the aim of the treatment is the delivery of the maximum tolerated dose, since this is considered the optimal dose to obtain clinical benefits in relation to the natural history of cancer. However, it is necessary that the intensity of the exercise guarantees adequate adaptation and recovery time; otherwise, strenuous exercise can be harmful. Experimental studies have shown that moderate exercise reduces the metastatic lung burden of transplanted liver cancer cells in mice, while strenuous exercise accelerates the growth of metastases [155].

Finally, there are contraindications to training [156], some similar to those of CR. Absolute contraindications are severe angina, dizziness or pre-syncope, cyanosis or O_2_ saturation at rest < 88% systolic blood pressure > 200 mm Hg or diastolic blood pressure > 110 mm Hg and basal heart rate above 120 bpm after two measurements five minutes apart. Other factors depend on the patient’s condition: low hemoglobin levels (<8.0 g/dl) are contraindications to high intensity protocols. There is an increased risk of bacterial infection in subjects with a low white blood cell count (<2000 and neutrophils < 1500) and if thrombocyte levels are <50,000, the risk of bleeding increases.

## 10. Exercise and Cancer Continuum

**Figure 3:**
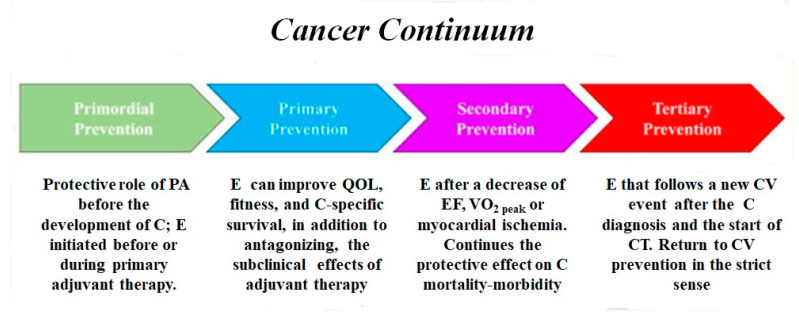
Exercise and cancer continuum, abbreviations as in the text.

The potential benefits of physical activity and exercise can occur throughout the natural history of CVD, as well of cancer [157]. In primordial prevention, cohort studies have shown that PA certainly has a protective role towards the development of at least some types of cancer [158,159,160]; furthermore, exercise training before or during primary adjuvant therapy can mitigate potential therapy-induced toxicity. In primary prevention, PA begins after the first chemotherapy/radiotherapy treatment and can improve QoL, fitness, and cancer survival, in addition to possibly antagonizing the effects of cancer therapy. In secondary prevention, exercise is prescribed after the detection of a decrease in the ejection fraction ≥ 10% (or beyond the lower limit of the normal values) of a VO_2peak_ < 15 mL/kg/min and myocardial ischemia [157]. Exercise continues the positive effect on functional capacity and fitness and QoL and reduces the fatigue and harmful effects of CT in advanced and metastatic cancer. CV prevention also begins: the cardiovascular continuum joins the cancer continuum (Figure 3). Finally, tertiary prevention occurs when exercise follows a new CV event (HF, acute coronary syndrome, stroke, or major arrhythmias) after cancer diagnosis and the initiation of chemotherapy/radiotherapy.

## 11. Limits and Future Perspectives

There are several gaps in the knowledge that make the optimal design of large-scale and randomized trials to test the definitive association between exercise and therapeutic effects in cancer difficult [161]. First, the existing evidence that supports the antitumor activity of exercise is limited mainly to observational data, and the clinical studies are limited. Thus, neither the dose nor the exercise prescription regimen can be derived accurately. Another confounding factor is represented by the individual factors related to the tumor and the host, both in the animal and in human models.

The opportunity to overcome these limitations could be represented by prospective studies based on short (4–6 weeks) preoperative exercise training, or longer exercise training (4–12 months) during adjuvant therapy or in patients with an advanced or metastatic stage of cancer, with different models of training (aerobic endurance vs. interval vs. resistance or combined; supervised vs. home vs. combined), and in large populations of well-defined types of cancer. Regarding the therapeutic and protective action of exercise on CV risk factors and CVD (coexisting or after cancer therapy), there is a wide demonstration of the efficacy of CR. Exercise could be a useful treatment, which is economic and without side effects, to improve the prognosis of cancer [161].

The concept of cancer pre-habilitation was recently introduced and is defined as a process of optimization of a patient’s physical and mental health (through exercise prescription and psychological and nutritional support), which occurs between the time of cancer diagnosis and the beginning of acute treatment [162,163]. It is being promoted as a way in which to improve the effectiveness of cancer treatment and survival. Cancer pre-habilitation has many points in common with or, in most cases, overlaps with CR.

## 12. Conclusions

The CR network represents the area in which the CORE model should be introduced in oncology. It is foreseeable that, with a few hours of specific education for operators, they will be able to acquire the basic oncological notions to start treating cancer patients. Cardiologists with cardio-oncological skills are the link between oncology and CR. The support and collaboration of oncologists will be indispensable, and the final result will be cultural growth within both disciplines. CORE, using the CR model, with a few additions, is a promising intervention for cancer survivors. Health policy makers should have a broader vision about the best and most cost-effective preventive interventions to reduce CV and oncologic mortality and should expand the entry to CR programs to include cancer patients.

## References

[1] Global Health Observatory (GHO) Data. https://www.who.int/gho/ncd/mortality_morbidity/en/.

[2] Curtin S.C. (2019). Trends in Cancer and Heart Disease Death Rates Among Adults Aged 45–64: United States, 1999–2017. Natl. Vital Stat. Rep..

[3] Van Dam R.M., Li T., Spiegelman D., Franco O.H., Hu F.B. (2008). Combined impact of lifestyle factors on mortality: Prospective cohort study in US women. BMJ.

[4] Koene R.J., Prizment A.E., Blaes A., Konety S.H. (2016). Shared Risk Factors in Cardiovascular Disease and Cancer. Circulation.

[5] Brown J.C., Caan B.J., Prado C.M., Weltzien E., Xiao J., Cespedes Feliciano E.M., Kroenke C.H., Meyerhardt J.A. (2019). Body Composition and Cardiovascular Events in Patients with Colorectal Cancer: A Population-Based Retrospective Cohort Study. JAMA Oncol..

[6] Gomes M.J., Martinez P.F., Pagan L.U., Damatto R.L., Cezar M.D.M., Lima A.R.L., Okoshi K., Politi M. (2017). Skeletal muscle aging: Influence of oxidative stress and physical exercise. Oncotarget.

[7] Iyengar N.M., Arthur R., Manson J.E., Chlebowski R.T., Kroenke C.H., Peterson L., Cheng T.D., Feliciano E.C., Lane D., Luo J. (2019). Association of Body Fat and Risk of Breast Cancer in Postmenopausal Women With Normal Body Mass Index: A Secondary Analysis of a Randomized Clinical Trial and Observational Study. JAMA Oncol..

[8] Hasin T., Gerber Y., Weston S.A., Jiang R., Killian J.M., Manemann S.M., Cerhan J.R., Roger V.L. (2016). Heart Failure After Myocardial Infarction Is Associated with Increased Risk of Cancer. JACC.

[9] Hasin T., Gerber Y., McNallan S.M., Weston S.A., Kushwaha S.S., Nelson T.J., Cerhan J.R., Roger V.L. (2013). Patients with heart failure have an increased risk of incident cancer. JACC.

[10] Banke A., Schou M., Videbaek L., Møller J.E., Torp-Pedersen C., Gustafsson F., Dahl J.S., Køber L., Hildebrandt P.R., Gislason G.H. (2016). Incidence of cancer in patients with chronic heart failure: A long-term follow-up study. Eur. J. Heart Fail..

[11] Oikawa T., Sakata Y., Nochioka K., Miura M., Abe R., Kasahara S., Sato M., Aoyanagi H., Shiroto T., Sugimura K. (2019). CHART-2 Investigators, Increased risk of cancer death in patients with chronic heart failure with a special reference to inflammation-A report from the CHART-2 Study. Intern. J. Cardiol..

[12] Farmakis D., Parissis J., Filippatos G. (2014). Insights into onco-cardiology: Atrial fibrillation in cancer. J. Am. Coll. Cardiol..

[13] Melloni C., Shrader P., Carver J., Piccini J.P., Thomas L., Fonarow G.C., Ansell J., Gersh B., Go A.S., Hylek E. (2017). ORBIT-AF Steering Committee, Management and outcomes of patients with atrial fibrillation and a history of cancer: The ORBIT-AF registry. Eur. Heart J. Qual. Care Clin. Outcomes.

[14] Conen D., Wong J.A., Sandhu R.K., Cook N.R., Lee I.M., Buring J.E., Albert C.M. (2016). Risk of Malignant Cancer Among Women with New-Onset Atrial Fibrillation. JAMA Cardiol..

[15] Lupo P.J., Schraw J.M., Desrosiers T.A., Nembhard W.N., Langlois P.H., Canfield M.A., Copeland G., Meyer R.E., Brown A.L., Chambers T.M. (2019). Association Between Birth Defects and Cancer Risk Among Children and Adolescents in a Population-Based Assessment of 10 Million Live Births. JAMA Oncol..

[16] Meijers W.C., de Boer R.A. (2019). Common risk factors for heart failure and cancer. Cardiovasc. Res..

[17] Miller K.D., Siegel R.L., Lin C.C., Mariotto A.B., Kramer J.L., Rowland J.H., Stein K.D., Alteri R., Jemal A. (2016). Cancer treatment and survivorship statistics. CA Cancer J. Clin..

[18] De Haas E.C., Oosting S.F., Lefrandt J.D., Wolffenbuttel B.H., Sleijfer D.T., Gietema J.A. (2010). The metabolic syndrome in cancer survivors. Lancet Oncol..

[19] Armenian S.H., Xu L., Ky B., Sun C., Farol L.T., Pal S.K., Douglas P.S., Bhatia S., Chao C. (2016). Cardiovascular Disease Among Survivors of Adult-Onset Cancer: A Community-Based Retrospective Cohort Study. J. Clin. Oncol..

[20] Chapman J.A., Meng D., Shepherd L., Parulekar W., Ingle J.N., Muss H.B., Palmer M., Yu C., Goss P.E. (2008). Competing causes of death from a randomized trial of extended adjuvant endocrine therapy for breast cancer. J. Natl. Cancer Inst..

[21] Simon M.S., Beebe-Dimmer J.L., Hastert T.A., Manson J.E., Cespedes Feliciano E.M., Neuhouser M.L., Ho G.Y.F., Freudenheim J.L., Strickler H., Ruterbusch J. (2018). Cardiometabolic risk factors and survival after breast cancer in the Women’s Health Initiative. Cancer.

[22] Rider J.R., Sandin F., Andrén O., Wiklund P., Hugosson J., Stattin P. (2013). Long-term outcomes among noncuratively treated men according to prostate cancer risk category in a nationwide, population-based study. Eur. Urol..

[23] Patnaik J.L., Byers T., Di Guiseppi C., Dabelea D., Denberg T.D. (2011). Cardiovascular disease competes with breast cancer as the leading cause of death for older females diagnosed with breast cancer’ A retrospective cohort study. Breast Cancer Res..

[24] Mulrooney D.A., Yeazel M.W., Kawashima T., Mertens A.C., Mitby P., Stovall M., Donaldson S.S., Green D.M., Sklar C.A., Robison L.L. (2009). Cardiac outcomes in a cohort of adult survivors of childhood and adolescent cancer: Retrospective analysis of the Childhood Cancer Survivor Study cohort. BMJ.

[25] Mulrooney D.A., Hyun G., Ness K.K., Ehrhardt M.J., Yasui Y., Duprez D., Howell R.M., Leisenring W.M., Constine L.S., Tonorezos E. (2020). Major cardiac events for adult survivors of childhood cancer diagnosed between 1970 and 1999: Report from the Childhood Cancer Survivor Study cohort. BMJ.

[26] Sturgeon K.M., Deng L., Bluethmann S.M., Zhou S., Trifiletti D.M., Jiang C., Kelly S.P., Zaorsky N.G. (2019). A population-based study of cardiovascular disease mortality risk in US cancer patients. Eur. Heart J..

[27] Ambrosetti M., Abreu A., Corrà U., Davos C.H., Hansen D., Frederix I., Iliou M.C., Pedretti R.F., Schmid J.P., Vigorito C. (2020). Secondary prevention through comprehensive cardiovascular rehabilitation: From knowledge to implementation, 2020 update, A position paper from the Secondary Prevention and Rehabilitation Section of the European Association of Preventive Cardiology. Eur. J. Prev. Cardiol..

[28] Pedretti R.F.E., Fattirolli F., Griffo R., Ambrosetti M., Angelino E., Brazzo S., Corrà U., Dasseni N., Faggiano P., Favretto G. (2018). Cardiac Prevention and Rehabilitation “3.0”: From acute to chronic phase; Position Paper of the ltalian Association for Cardiovascular Prevention and Rehabilitation (GICR-IACPR). Monaldi Arch. Chest. Dis..

[29] Vitale G., Romano G., Di Franco A., Caccamo G., Nugara C., Ajello L., Storniolo S., Sarullo S., Agnese V., Giallauria F. (2019). Early Effects of Sacubitril/Valsartan on Exercise Tolerance in Patients with Heart Failure with Reduced Ejection Fraction. J. Clin. Med..

[30] Giallauria F., Cirillo P., Lucci R., Pacileo M., De Lorenzo A., D’Agostino M., Moschella S., Psaroudaki M., Del Forno D., Orio F. (2008). Left ventricular remodeling in patients with moderate systolic dysfunction after myocardial infarction: Favorable effects of exercise training and predictive role of N-terminal pro-brain natriuretic peptide. Eur. J. Cardiovasc. Prev. Rehabil..

[31] Giallauria F., Galizia G., Lucci R., D’Agostino M., Vitelli A., Maresca L., Orio F., Vigorito C. (2009). Favorable effects of exercise-based cardiac rehabilitation after acute myocardial infarction on left atrial remodeling. Int. J. Cardiol..

[32] Giallauria F., Lucci R., D’Agostino M., Vitelli A., Maresca L., Mancini M., Aurino M., Del Forno D., Giannuzzi P., Vigorito C. (2009). Two year multi-comprehensive secondary prevention program: Favourable effects on cardiovascular functional capacity and coronary risk profile after acute myocardial infarction. J. Cardiovasc. Med. Hagerstown.

[33] Giannuzzi P., Temporelli P.L., Marchioli R., Maggioni A.P., Balestroni G., Ceci V., Chieffo C., Gattone M., Griffo R., Schweiger C. (2008). GOSPEL Investigators Global Secondary Prevention Strategies to Limit Event Recurrence after Myocardial Infarction. Arch. Intern. Med..

[34] Giallauria F., Acampa W., Ricci F., Vitelli A., Maresca L., Mancini M., Grieco A., Gallicchio R., Xhoxhi E., Spinelli L. (2012). Effects of exercise training started within 2 weeks after acute myocardial infarction on myocardial perfusion and left ventricular function: A gated SPECT imaging study. Eur. J. Prev. Cardiol..

[35] Giallauria F., Acampa W., Ricci F., Vitelli A., Torella G., Lucci R., Del Prete G., Zampella E., Assante R., Rengo G. (2013). Exercise training early after acute myocardial infarction reduces stress-induced hypoperfusion and improves left ventricular function. Eur. J. Nucl. Med. Mol. Imaging.

[36] Tarro Genta F., Eleuteri E., Temporelli P.L., Comazzi F., Tidu M., Bouslenko Z., Bertolin F., Vigorito C., Giannuzzi P., Giallauria F. (2013). Flow-mediated dilation normalization predicts outcome in chronic heart failure patients. J. Cardiac. Fail..

[37] Giallauria F., Cirillo P., D’Agostino M., Petrillo G., Vitelli A., Pacileo M., Angri V., Chiariello M., Vigorito C. (2011). Effects of exercise training on high-mobility group box-1 levels after acute myocardial infarction. J. Cardiac. Fail..

[38] Cirillo P., Giallauria F., Pacileo M., Petrillo G., D’Agostino M., Vigorito C., Chiariello M. (2009). Increased High Mobility Group Box-1 Protein levels are associated with impaired cardiopulmonary and echocardiographic findings after acute myocardial infarction. J. Card. Fail..

[39] Giallauria F., De Lorenzo A., Pilerci F., Manakos A., Lucci R., Psaroudaki M., D’Agostino M., Del Forno D., Vigorito C. (2006). Long-Term Effects of Cardiac Rehabilitation on End-Exercise Heart Rate Recovery after Myocardial Infarction. Eur. J. Cardiovasc. Prev. Rehabil..

[40] Giallauria F., Lucci R., Pietrosante M., Gargiulo G., De Lorenzo A., D’Agostino M., Gerundo G., Abete P., Rengo F., Vigorito C. (2006). Exercise-based Cardiac Rehabilitation improves Heart Rate Recovery in Elderly Patients after Acute Myocardial Infarction. J. Gerontol. Ser. A Biol. Sci. Med. Sci..

[41] Giallauria F., De Lorenzo A., Pilerci F., Manakos A., Lucci R., Psaroudaki M., D’Agostino M., Del Forno D., Vigorito C. (2006). Reduction of NT-pro-BNP levels with exercise-based cardiac rehabilitation in patients with left ventricular dysfunction after myocardial infarction. Eur. J. Cardiovasc. Prev. Rehabil..

[42] Giallauria F., Lucci R., De Lorenzo A., D’Agostino M., Del Forno D., Vigorito C. (2006). Favourable effects of exercise training on N-terminal pro-brain natriuretic peptide plasma levels in elderly patients after acute myocardial infarction. Age Ageing.

[43] Smart N., Meyer T., Butterfield J.A., Faddy S.C., Passino C., Malfatto G., Jonsdottir S., Sarullo F., Wisloff U., Vigorito C. (2012). Individual patient meta-analysis of exercise training effects on systemic brain natriuretic peptide expression in heart failure. Eur. J. Prev. Cardiol..

[44] Dieberg G., Ismail H., Giallauria F., Smart N.A. (2015). Clinical outcomes and cardiovascular responses to exercise training in heart failure patients with preserved ejection fraction: A systematic review and meta-analysis. J. Appl. Physiol. 1985.

[45] Smart N.A., Giallauria F., Dieberg G. (2013). Efficacy of inspiratory muscle training in chronic heart failure patients: A systematic review and meta-analysis. Int. J. Cardiol..

[46] Smart N.A., Dieberg G., Giallauria F. (2013). Functional electrical stimulation for chronic heart failure: A meta-analysis. Int. J. Cardiol..

[47] Smart N.A., Dieberg G., Giallauria F. (2013). Intermittent versus continuous exercise training in chronic heart failure: A meta-analysis. Int. J. Cardiol..

[48] Vigorito C., Giallauria F. (2014). Effects of exercise on cardiovascular performance in the elderly. Front. Physiol..

[49] Schmid D., Leitzmann M.F. (2014). Association between physical activity and mortality among breast cancer and colo-rectal cancer survivors: A systematic review and meta-analysis. Ann. Oncol..

[50] Lahart I.M., Metsios G.S., Nevill A.M., Carmichael A.R. (2015). Physical activity, risk of death and recurrence in breast cancer survivors: A systematic review and meta-analysis of epidemiological studies. Acta Oncol..

[51] Bullard T., Ji M., An R., Trinh L., Mackenzie M., Mullen S.P. (2019). A systematic review and meta-analysis of adherence to physical activity interventions among three chronic conditions: Cancer, cardiovascular disease, and diabetes. BMC Public Health.

[52] Buffart L.M., Kalter J., Sweegers M.G., Courneya K.S., Newton R.U., Aaronson N.K., Jacobsen P.B., May A.M., Galvão D.A., Chinapaw M.J. (2017). Effects and moderators of exercise on quality of life and physical function in patients with cancer: An individual patient data meta-analysis of 34 RCTs. Cancer Treat. Rev..

[53] Buffart L.M., Sweegers M.G., May A.M., Chinapaw M.J., van Vulpen J.K., Newton R.U., Galvão D.A., Aaronson N.K., Stuiver M.M., Jacobsen P.B. (2018). Targeting Exercise Interventions to Patients With Cancer in Need: An Individual Patient Data Meta-Analysis. J. Natl. Cancer Inst..

[54] Morales J.S., Valenzuela P.L., Rincón-Castanedo C., Takken T., Fiuza-Luces C., Santos-Lozano A., Lucia A. (2018). Exercise training in childhood cancer: A systematic review and meta-analysis of randomized controlled trials. Cancer Treat. Rev..

[55] Jones L.W., Courneya K.S., Mackey J.R., Muss H.B., Pituskin E.N., Scott J.M., Hornsby W.E., Coan A.D., Herndon J.E., Douglas P.S. (2012). Cardiopulmonary function and age-related decline across the breast cancer survivorship continuum. J. Clin. Oncol..

[56] Jones L.W., Liang Y., Pituskin E.N., Battaglini C.L., Scott J.M., Hornsby W.E., Haykowsky M. (2011). Effect of exercise training on peak oxygen consumption in patients with cancer: A meta-analysis. Oncologist.

[57] Pugliese N.R., Masi S. (2020). The emerging role of endothelial function in cardiovascular oncology. Eur. J. Prev. Cardiol..

[58] Toya T., Sara J.D., Corban M.T., Taher R., Godo S., Herrmann J., Lerman L.O., Lerman A. (2020). Assessment of peripheral endothelial function predicts future risk of solid-tumor cancer. Eur. J. Prev. Cardiol..

[59] Giallauria F., Vitelli A., Maresca L., Santucci De Magistris M., Chiodini P., Mattiello A., Gentile M., Mancini M., Grieco A., Russo A. (2016). Exercise training improves cardiopulmonary and endothelial function in women with breast cancer: Findings from the Diana-5 dietary intervention study. Intern. Emerg. Med..

[60] Giallauria F., Vitelli A., Maresca L., Santucci De Magistris M., Chiodini P., Mattiello A., Gentile M., Mancini M., Grieco A., Russo A. (2015). Exercise training improves heart rate recovery in women with breast cancer. Springerplus.

[61] Van Waart H., Stuiver M.M., van Harten W.H., Geleijn E., Kieffer J.M., Buffart L.M., de Maaker-Berkhof M., Boven E., Schrama J., Geenen M.M. (2015). Effect of Low-Intensity Physical Activity and Moderate- to High-Intensity Physical Exercise During Adjuvant Chemotherapy on Physical Fitness, Fatigue, and Chemotherapy Completion Rates: Results of the PACES Randomized Clinical Trial. J. Clin. Oncol..

[62] Hornsby W.E., Douglas P.S., West M.J., Kenjale A.A., Lane A.R., Schwitzer E.R., Ray K.A., Herndon J.E., Coan A., Gutierrez A. (2014). Safety and efficacy of aerobic training in operable breast cancer patients receiving neoadjuvant chemotherapy: A phase II randomized trial. Acta Oncol..

[63] Segal R.J., Reid R.D., Courneya K.S., Sigal R.J., Kenny G.P., Prud’Homme D.G., Malone S.C., Wells G.A., Scott C.G., Slovinec D’Angelo M.E. (2009). Randomized controlled trial of resistance or aerobic exercise in men receiving radiation therapy for prostate cancer. J. Clin. Oncol..

[64] Taaffe D.R., Newton R.U., Spry N., Joseph D., Chambers S.K., Gardiner R.A., Wall B.A., Cormie P., Bolam K.A., Galvão D.A. (2017). Effects of Different Exercise Modalities on Fatigue in Prostate Cancer Patients Undergoing Androgen Deprivation Therapy: A Year-long Randomised Controlled Trial. Eur. Urol..

[65] Thomas G.A., Cartmel B., Harrigan M., Fiellin M., Capozza S., Zhou Y., Ercolano E., Gross C.P., Hershman D., Ligibel J. (2017). The effect of exercise on body composition and bone mineral density in breast cancer survivors taking aromatase inhibitors. Obes. Silver Spring.

[66] Courneya K.S., Sellar C.M., Stevinson C., McNeely M.L., Peddle C.J., Friedenreich C.M., Tankel K., Basi S., Chua N., Mazurek A. (2009). Randomized controlled trial of the effects of aerobic exercise on physical functioning and quality of life in lymphoma patients. J. Clin. Oncol..

[67] Cheville A.L., Moynihan T., Herrin J., Loprinzi C., Kroenke K. (2019). Effect of Collaborative Telerehabilitation on Functional Impairment and Pain Among Patients with Advanced-Stage Cancer: A Randomized Clinical Trial. JAMA Oncol..

[68] Scott J.M., Iyengar N.M., Nilsen T.S., Michalski M., Thomas S.M., Herndon J., Sasso J., Yu A., Chandarlapaty S., Dang C.T. (2018). Feasibility, safety, and efficacy of aerobic training in pretreated patients with metastatic breast cancer: A randomized controlled trial. Cancer.

[69] Howden E.J., Bigaran A., Beaudry R., Fraser S., Selig S., Foulkes S., Antill Y., Nightingale S., Loi S., Haykowsky M.J. (2019). Exercise as a diagnostic and therapeutic tool for the prevention of cardiovascular dysfunction in breast cancer patients. Eur. J. Prev. Cardiol..

[70] Costello B.T., Roberts T.J., Howden E.J., Bigaran A., Foulkes S.J., Beaudry R.I., Janssens K., Haykowsky M.J., Antill Y., Nightingale S. (2019). Exercise attenuates cardiotoxicity of anthracycline chemotherapy measured by global longitudinal strain. JACC Car. Oncol..

[71] Jones L.W., Douglas P.S., Khouri M.G., Mackey J.R., Wojdyla D., Kraus W.E., Whellan D.J., O’Connor C.M. (2014). Safety and efficacy of aerobic training in patients with cancer who have heart failure: An analysis of the HF-ACTION randomized trial. J. Clin. Oncol..

[72] Courneya K.S., Segal R.J., Gelmon K., Mackey J.R., Friedenreich C.M., Yasui Y., Reid R.D., Proulx C., Trinh L., Dolan L.B. (2014). Predictors of adherence to different types and doses of supervised exercise during breast cancer chemotherapy. Int. J. Behav. Nutr. Phys. Act..

[73] Witlox L., Hiensch A.E., Velthuis M.J., Steins Bisschop C.N., Los M., Erdkamp F.L.G., Bloemendal H.J., Verhaar M., Ten Bokkel Huinink D., van der Wall E. (2018). Four-year effects of exercise on fatigue and physical activity in patients with cancer. BMC Med..

[74] Van Blarigan E.L., Fuchs C.S., Niedzwiecki D., Zhang S., Saltz L.B., Mayer R.J., Mowat R.B., Whittom R., Hantel A., Benson A. (2018). Association of Survival With Adherence to the American Cancer Society Nutrition and Physical Activity Guidelines for Cancer Survivors After Colon Cancer Diagnosis: The CALGB 89803/Alliance Trial. JAMA Oncol..

[75] Giallauria F., Gentile M., Chiodini P., Berrino F., Mattiello A., Maresca L., Vitelli A., Mancini M., Grieco A., Lucci R. (2014). Exercise training reduces high mobility group box-1 protein levels in women with breast cancer: Findings from the DIANA-5 study. Monaldi Arch. Chest Dis..

[76] Repka C.P., Hayward R. (2016). Oxidative Stress and Fitness Changes in Cancer Patients after Exercise Training. Med. Sci. Sports Exerc..

[77] Repka C.P., Hayward R. (2018). Effects of an Exercise Intervention on Cancer-Related Fatigue and Its Relationship to Markers of Oxidative Stress. Integr. Cancer Ther..

[78] Ashcraft K.A., Warner A.B., Jones L.W., Dewhirst M.W. (2019). Exercise as Adjunct Therapy in Cancer. Semin. Radiat. Oncol..

[79] Betof A.S., Lascola C.D., Weitzel D., Landon C., Scarbrough P.M., Devi G.R., Palmer G., Jones L.W., Dewhirst M.W. (2015). Modulation of murine breast tumor vascularity, hypoxia and chemotherapeutic response by exercise. J. Natl. Cancer Inst..

[80] Shalamzari S.A., Agha-Alinejad H., Alizadeh S., Shahbazi S., Khatib Z.K., Kazemi A., Saei M.A., Minayi N. (2014). The effect of exercise training on the level of tissue IL-6 and vascular endothelial growth factor in breast cancer bearing mice. Iran J. Basic Med. Sci..

[81] Jones L.W., Antonelli J., Masko E.M., Broadwater G., Lascola C.D., Fels D., Dewhirst M.W., Dyck J.R.B., Nagendran J., Flores C.T. (2012). Exercise modulation of the host-tumor interaction in an orthotopic model of murine prostate cancer. J. Appl. Physiol. 1985.

[82] Schadler K.L., Thomas N.J., Galie P.A., Bhang D.H., Roby K.C., Addai P., Till J.E., Sturgeon K., Zaslavsky A., Chen C.S. (2016). Tumor vessel normalization after aerobic exercise enhances chemotherapeutic efficacy. Oncotarget.

[83] Jones L.W., Fels D.R., West M., Allen J.D., Broadwater G., Barry W.T., Wilke L.G., Masko E., Douglas P.S., Dash R.C. (2013). Modulation of circulating angiogenic factors and tumor biology by aerobic training in breast cancer patients receiving neoadjuvant chemotherapy. Cancer Prev. Res. Phila.

[84] Schumann M., Schulz H., Hackney A.C., Bloch W. (2017). Feasibility of high-intensity interval training with hyperoxia vs. intermittent hyperoxia and hypoxia in cancer patients undergoing chemotherapy—Study protocol of a randomized controlled trial. Contemp. Clin. Trials Commun..

[85] Freitag N., Weber P.D., Sanders T.C., Schulz H., Bloch W., Schumann M. (2018). High-intensity interval training and hyperoxia during chemotherapy: A case report about the feasibility, safety and physical functioning in a colorectal cancer patient. Med. Baltim..

[86] Dewhirst M.W., Secomb T.W. (2017). Transport of drugs from blood vessels to tumour tissue. Nat. Rev. Cancer..

[87] Evans E.S., Hackney A.C., McMurray R.G., Randell S.H., Muss H.B., Deal A.M., Battaglini C.L. (2015). Impact of Acute Intermittent Exercise on Natural Killer Cells in Breast Cancer Survivors. Integr. Cancer Ther..

[88] Schmidt T., Hermes A., Weisser B. (2017). Physical Training Influences the Immune System of Breast Cancer Patients. Dtsch. Z. Sportmed..

[89] Bacurau A.V., Belmonte M.A., Navarro F., Moraes M.R., Pontes F.L., Pesquero J.L., Arauju R.C., Pereira Bacurau R.F. (2007). Effect of a high-intensity exercise training on the metabolism and function of macrophages and lymphocytes of walker 256 tumor bearing rats. Exp. Biol. Med. Maywood.

[90] Li L., Liu H., Du L., Xi P., Wang Q., Li Y., Liu D. (2018). MiR-449a Suppresses LDHA-Mediated Glycolysis to Enhance the Sensitivity of Non-Small Cell Lung Cancer Cells to Ionizing Radiation. Oncol. Res..

[91] Okwuosa T.M., Ray R.M., Palomo A., Foraker R.E., Johnson L., Paskett E.D., Caan B., Jones L.W. (2019). Pre-Diagnosis Exercise and Cardiovascular Events in Primary Breast Cancer. JACC CardioOncol..

[92] Jones L.W., Liu Q., Armstrong G.T., Ness K.K., Yasui Y., Devine K., Tonorezos E., Soares-Miranda L., Sklar C.A., Douglas P.S. (2014). Exercise and risk of major cardiovascular events in adult survivors of childhood Hodgkin lymphoma: A report from the childhood cancer survivor study. J. Clin. Oncol..

[93] Jones L.W., Habel L.A., Weltzien E., Castillo A., Gupta D., Kroenke C.H., Kwan M.L., Quesenberry C.P., Scott J., Sternfeld B. (2016). Exercise and Risk of Cardiovascular Events in Women With Nonmetastatic Breast Cancer. J. Clin. Oncol..

[94] Adams S.C., DeLorey D.S., Davenport M.H., Stickland M.K., Fairey A.S., North S., Szczotka A., Courneya K.S. (2017). Effects of high-intensity aerobic interval training on cardiovascular disease risk in testicular cancer survivors: A phase 2 randomized controlled trial. Cancer.

[95] Lee K., Tripathy D., Demark-Wahnefried W., Courneya K.S., Sami N., Bernstein L., Spicer D., Buchanan T.A., Mortimer J.E., Dieli-Conwright C.M. (2019). Effect of Aerobic and Resistance Exercise Intervention on Cardiovascular Disease Risk in Women With Early-Stage Breast Cancer: A Randomized Clinical Trial. JAMA Oncol..

[96] Canale M.L., Camerini A., Huqi A., Lilli A., Bisceglia I., Parrini I., Lestuzzi C., Del Meglio J., Donati S., Venturini E. (2019). Cardiovascular Risk Factors and Timing of Anthracyclines and Trastuzumab Cardiac Toxicity. Anticancer Res..

[97] Dolinsky V.W., Rogan K.J., Sung M.M., Zordoky B.N., Haykowsky M.J., Young M.E., Jones L.W., Dyck J.R. (2013). Both aerobic exercise and resveratrol supplementation attenuate doxorubicin-induced cardiac injury in mice. Am. J. Physiol. Endocrinol. Metab..

[98] Jones L., Dolinsky V.W., Haykowsky M.J.F., Pattreson I., Allen J.D., Scott J.M., Rogan K., Khouri M., Hornsby W., Young M. Effects of aerobic training to improve cardiovascular function and prevent cardiac remodeling after cytotoxic therapy in early breast cancer. Proceedings of the 102nd Annual Meeting of the American Association of Cancer Research.

[99] Kavazis A.N., Smuder A.J., Min K., Tümer N., Powers S.K. (2010). Short-term exercise training protects against doxorubicin-induced cardiac mitochondrial damage independent of HSP72. Am. J. Physiol Heart Circ. Physiol..

[100] Scott J.M., Khakoo A., Mackey J.R., Haykowsky M.J., Douglas P.S., Jones L.W. (2011). Modulation of anthracycline-induced cardiotoxicity by aerobic exercise in breast cancer: Current evidence and underlying mechanisms. Circulation.

[101] Werner C., Hanhoun M., Widmann T., Kazakov A., Semenov A., Pöss J., Bauersachs J., Thum T., Pfreundschuh M., Müller P. (2008). Effects of physical exercise on myocardial telomere-regulating proteins, survival pathways, and apoptosis. J. Am. Coll. Cardiol..

[102] Boström P., Mann N., Wu J., Quintero P.A., Plovie E.R., Panáková D., Gupta R.K., Xiao C., MacRae C.A., Rosenzweig A. (2010). C/EBPβ controls exercise-induced cardiac growth and protects against pathological cardiac remodeling. Cell.

[103] French J.P., Hamilton K.L., Quindry J.C., Lee Y., Upchurch P.A., Powers S.K. (2008). Exercise-induced protection against myocardial apoptosis and necrosis: MnSOD, calcium-handling proteins, and calpain. FASEB J..

[104] Weikel K.A., Ruderman N.B., Cacicedo J.M. (2016). Unraveling the actions of AMP-activated protein kinase in metabolic diseases: Systemic to molecular insights. Metabolism.

[105] Kolibaba K.S., Druker B.J. (1997). Protein tyrosine kinases and cancer. Biochim. Biophys. Acta.

[106] Scott J.M., Lakoski S., Mackey J.R., Douglas P.S., Haykowsky M.J., Jones L.W. (2013). The potential role of aerobic exercise to modulate cardiotoxicity of molecularly targeted cancer therapeutics. Oncologist.

[107] Lebrasseur N.K., Coté G.M., Miller T.A., Fielding R.A., Sawyer D.B. (2003). Regulation of neuregulin/ErbB signaling by contractile activity in skeletal muscle. Am. J. Physiol. Cell Physiol..

[108] McMullen J.R., Amirahmadi F., Woodcock E.A., Schinke-Braun M., Bouwman R.D., Hewitt K.A., Mollica J.P., Zhang L., Zhang Y., Shioi T. (2007). Protective effects of exercise and phosphoinositide 3-kinase (p110alpha) signaling in dilated and hypertrophic cardiomyopathy. Proc. Natl. Acad. Sci. USA.

[109] Haykowsky M.J., Mackey J.R., Thompson R.B., Jones L.W., Paterson D.I. (2009). Adjuvant trastuzumab induces ventricular remodeling despite aerobic exercise training. Clin. Cancer Res..

[110] Muñoz-Chápuli R., Quesada A.R., Angel Medina M. (2004). Angiogenesis and signal transduction in endothelial cells. Cell Mol. Life Sci..

[111] Lin J., Wu H., Tarr P.T., Zhang C.Y., Wu Z., Boss O., Michael L.F., Puigserver P., Isotani E., Olson E.N. (2002). Transcriptional co-activator PGC-1 alpha drives the formation of slow-twitch muscle fibres. Nature.

[112] Kunisada K., Negoro S., Tone E., Funamoto M., Osugi T., Yamada S., Okabe M., Kishimoto T., Yamauchi-Takihara K. (2000). Signal transducer and activator of transcription 3 in the heart transduces not only a hypertrophic signal but a protective signal against doxorubicin-induced cardiomyopathy. Proc. Natl. Acad. Sci. USA.

[113] Groarke J.D., Tanguturi V.K., Hainer J., Klein J., Moslehi J.J., Ng A., Forman D.E., Di Carli M.F., Nohria A. (2015). Abnormal exercise response in long-term survivors of hodgkin lymphoma treated with thoracic irradiation: Evidence of cardiac autonomic dysfunction and impact on outcomes. J. Am. Coll Cardiol..

[114] Scott J.M., Jones L.W., Hornsby W.E., Koelwyn G.J., Khouri M.G., Joy A.A., Douglas P.S., Lakoski S.G. (2014). Cancer therapy-induced autonomic dysfunction in early breast cancer: Implications for aerobic exercise training. Int. J. Cardiol..

[115] Lee D.H., Park S., Lim S.M., Lee M.K., Giovannucci E.L., Kim J.H., Kim S.I., Jeon J.Y. (2016). Resting heart rate as a prognostic factor for mortality in patients with breast cancer. Breast Cancer Res. Treat..

[116] Park J., Kim J.H., Park Y., Park S.J., Cheon J.H., Kim W.H., Park J.S., Jeon J.Y., Kim T.I. (2018). Resting heart rate is an independent predictor of advanced colorectal adenoma recurrence. PLoS ONE.

[117] Lakoski S.G., Jones L.W., Krone R.J., Stein P.K., Scott J.M. (2015). Autonomic dysfunction in early breast cancer: Incidence, clinical importance, and underlying mechanism. Am. Heart J..

[118] Cramer L., Hildebrandt B., Kung T., Wichmann K., Springer J., Doehner W., Sandek A., Valentova M., Stojakovic T., Scharnagl H. (2014). Cardiovascular function and predictors of exercise capacity in patients with colorectal cancer. J. Am. Coll. Cardiol..

[119] Jolly M.A., Brennan D.M., Cho L. (2011). Impact of exercise on heart rate recovery. Circulation.

[120] Niederer D., Vogt L., Thiel C., Schmidt K., Bernhörster M., Lungwitz A., Jäger E., Banzer W. (2013). Exercise effects on HRV in cancer patients. Int. J. Sports Med..

[121] Scott J.M., Nilsen T.S., Gupta Det Jones L.W. (2018). Exercise Therapy and Cardiovascular Toxicity in Cancer. Circulation.

[122] Gilchrist S.C., Barac A., Ades P.A., Alfano C.M., Franklin B.A., Jones L.W., La Gerche A., Ligibel J.A., Lopez G., Madan K. (2019). Cardio-Oncology Rehabilitation to Manage Cardiovascular Outcomes in Cancer Patients and Survivors: A Scientific Statement From the American Heart Association. Circulation.

[123] Dittus K.L., Lakoski S.G., Savage P.D., Kokinda N., Toth M., Stevens D., Woods K., O’Brien P., Ades P.A. (2015). Exercise-Based Oncology Rehabilitation: Leveraging the Cardiac Rehabilitation Model. J. Cardiopulm. Rehabil. Prev..

[124] Fowler M.E., Akinyemiju T.F. (2017). Meta-analysis of the association between dietary inflammatory index (DII) and cancer outcomes. Int. J. Cancer..

[125] Fan Y., Jin X., Man C., Gao Z., Wang X. (2017). Meta-analysis of the association between the inflammatory potential of diet and colorectal cancer risk. Oncotarget.

[126] Tabung F.K., Liu L., Wang W., Fung T.T., Wu K., Smith-Warner S.A., Cao Y., Hu F.B., Ogino S., Fuchs C.S. (2018). Association of Dietary Inflammatory Potential With Colorectal Cancer Risk in Men and Women. JAMA Oncol..

[127] Moradi S., Issah A., Mohammadi H., Mirzaei K. (2018). Associations between dietary inflammatory index and incidence of breast and prostate cancer: A systematic review and meta-analysis. Nutrition.

[128] Sofi F., Dinu M., Pagliai G., Cesari F., Gori A.M., Sereni A., Becatti M., Fiorillo C., Marcucci R., Casini A. (2018). Low-Calorie Vegetarian Versus Mediterranean Diets for Reducing Body Weight and Improving Cardiovascular Risk Profile: CARDIVEG Study (Cardiovascular Prevention With Vegetarian Diet). Circulation.

[129] Toledo E., Salas-Salvadó J., Donat-Vargas C., Buil-Cosiales P., Estruch R., Ros E., Corella D., Fitó M., Hu F.B., Arós F. (2015). Mediterranean Diet and Invasive Breast Cancer Risk Among Women at High Cardiovascular Risk in the PREDIMED Trial: A Randomized Clinical Trial. JAMA Intern. Med..

[130] Soldati L., Di Renzo L., Jirillo E., Ascierto P.A., Marincola F.M., De Lorenzo A. (2018). The influence of diet on anti-cancer immune responsiveness. J. Transl. Med..

[131] Demark-Wahnefried W., Rogers L.Q., Alfano C.M., Thomson C.A., Courneya K.S., Meyerhardt J.A., Stout N.L., Kvale E., Ganzer H., Ligibel J.A. (2015). Practical Clinical Interventions for Diet, Physical Activity, and Weight Control in Cancer Survivors. CA Cancer J. Clin..

[132] Tollosa D.N., Tavener M., Hure A., James E.L. (2019). Adherence to multiple health behaviours in cancer survivors: A systematic review and meta-analysis. J. Cancer Surviv..

[133] Baguley B.J., Bolam K.A., Wright O.R.L., Skinner T.L. (2017). The Effect of Nutrition Therapy and Exercise on Cancer-Related Fatigue and Quality of Life in Men With Prostate Cancer: A Systematic Review. Nutrients.

[134] Kwan M.L., Weltzien E., Kushi L.H., Castillo A., Slattery M.L., Caan B.J. (2009). Dietary patterns and breast cancer recurrence and survival among women with early-stage breast cancer. J. Clin. Oncol..

[135] Blackburn G.L., Wang K. (2007). A Dietary Fat Reduction and Breast Cancer Outcome: Results From the Women’s Intervention Nutrition Study (WINS). Am. J. Clin. Nutr..

[136] Inoue-Choi M., Robien K., Lazovich D. (2013). Adherence to the WCRF/AICR guidelines for cancer prevention is associated with lower mortality among older female cancer survivors. Cancer Epidemiol. Biomark. Prev..

[137] Westerink N.L., Nuver J., Lefrandt J.D., Vrieling A.H., Gietema J.A., Walencamp A.M.E. (2016). Cancer treatment induced metabolic syndrome: Improving outcome with lifestyle. Crit Rev. Oncol Hematol..

[138] Bellastella G., Scappaticcio L., Esposito K., Giugliano D., Maiorino M.I. (2018). Metabolic Syndrome and Cancer: “The Common Soil Hypothesis”. Diabetes Res. Clin. Pract..

[139] Darzy K.H., Shalet S.M. (2009). Hypopituitarism following radiotherapy. Pituitary.

[140] Casco S., Soto-Vega E. (2016). Development of metabolic syndrome associated to cancer therapy: Review. Horm. Cancer.

[141] Haugnes H.S., Aass N., Fossa S.D., Klepp O., Wist E.A., Svartberg J., Wilsgaard T., Bremnes R.M. (2007). Components of the metabolic syndrome in long-term survivors of testicular cancer. Ann. Oncol..

[142] Baker K.S., Chow E.J., Goodman P.J., Leisenring W.M., Dietz A.C., Perkins J.L., Chow L., Sinaiko A., Moran A., Petryk A. (2013). Impact of treatment exposures on cardiovascular risk and insulin resistance in childhood cancer survivors. Cancer Epidemiol. Biomark. Prev..

[143] Rosen G.P., Nguyen H.T., Shaibi G.Q. (2013). Metabolic syndrome in pediatric cancer survivors mechanistic review. Pediatr. Blood Cancer.

[144] Floyd J., Mirza I., Sachs B., Perry M.C. (2006). Hepatotoxicity of chemotherapy. Semin. Oncol..

[145] Gibson T.M., Ehrhardt M.J., Ness K.K. (2016). Obesity and metabolic syndrome among adult survivors of childhood leukemia. Curr Treat. Options Oncol..

[146] Ekblom Ö., Ekblom-Bak E., Rosengren A., Hallsten M., Bergstrom G., Borjesson M. (2015). Cardiorespiratory fitness, sedentary behaviour and physical activity are independently associated with the metabolic syndrome, results from theSCAPIS pilot study. PLoS ONE.

[147] Canale M.L., Camerini A., Casolo G., Lilli A., Bisceglia I., Parrini I., Lestuzzi C., Del Meglio J., Puccetti C., Camerini L. (2020). Incidence of Pericardial Effusion in Patients With Advanced Non-Small Cell Lung Cancer Receiving Immunotherapy. Adv. Ther..

[148] Schmitz K.H., Troxel A.B., Dean L.T., DeMichele A., Brown J.C., Sturgeon K., Zhang Z., Evangelisti M., Spinelli B., Kallan M.J. (2019). Effect of Home-Based Exercise and Weight Loss Programs on Breast Cancer-Related Lymphedema Outcomes Among Overweight Breast Cancer Survivors: The WISER Survivor Randomized Clinical Trial. JAMA Oncol..

[149] Jones L.W., Eves N.D., Scott J.M. (2017). Bench-to-Bedside Approaches for Personalized Exercise Therapy in Cancer. Am. Soc. Clin. Oncol. Educ. Book.

[150] Holmes M.D., Chen W.Y., Feskanich D., Kroenke C.H., Colditz G.A. (2005). Physical activity and survival after breast cancer diagnosis. JAMA.

[151] Morikawa T., Kuchiba A., Yamauchi M., Meyerhardt J.A., Shima K., Nosho K., Chan A.T., Giovannucci E., Fuchs C.S., Ogino S. (2011). Association of CTNNB1 (betacatenin) alterations, body mass index, and physical activity with survival in patients with colorectal cancer. JAMA.

[152] Nkondjock A., Robidoux A., Paredes Y., Narod S.A., Ghadirian P. (2006). Diet, lifestyle and BRCA related breast cancer risk among French-Canadians. Breast Cancer Res. Treat..

[153] Courneya K.S., McKenzie D.C., Mackey J.R., Gelmon K., Friedenreich C.M., Yasui Y., Reid R.D., Vallerand J.R., Adams S.C., Proulx C. (2014). Subgroup effects in a randomised trial of different types and doses of exercise during breast cancer chemotherapy. Br. J. Cancer.

[154] Zimmer P., Trebing S., Timmers-Trebing U., Schenk A., Paust R., Bloch W., Rudolph R., Streckmann F., Baumann F.T. (2018). Eight-week, multimodal exercise counteracts a progress of chemotherapy-induced peripheral neuropathy and improves balance and strength in metastasized colorectal cancer patients: A randomized controlled trial. Support. Care Cancer.

[155] Hong F., Ye W., Kuo C.H., Zhang Y., Qian Y., Korivi M. (2019). Exercise Intervention Improves Clinical Outcomes, but the “Time of Session” is Crucial for Better Quality of Life in Breast Cancer Survivors: A Systematic Review and Meta-Analysis. Cancers Basel.

[156] Zhang Q.B., Zhang B.H., Zhang K.Z., Meng X.T., Jia Q.A., Zhang Q.B., Bu Y., Zhu X.D., Ma D.N., Ye B.G. (2016). Moderate swimming suppressed the growth and metastasis of the transplanted liver cancer in mice model: With reference to nervous system. Oncogene.

[157] D’Ascenzi F., Anselmi F., Fiorentini C., Mannucci R., Bonifazi M., Mondillo S. (2019). The benefits of exercise in cancer patients and the criteria for exercise prescription in cardio-oncology. Eur. J. Prev. Cardiol..

[158] Scott J.M., Adams S.C., Koelwyn G.J., Jones L.W. (2016). Cardiovascular Late Effects and Exercise Treatment in Breast Cancer: Current Evidence and Future Directions. Can. J. Cardiol..

[159] Mok A., Khaw K.T., Luben R., Wareham N., Brage S. (2019). Physical activity trajectories and mortality: Population based cohort study. BMJ.

[160] Saint-Maurice P.F., Coughlan D., Kelly S.P., Keadle S.K., Cook M.B., Carlson S.A., Fulton J.E., Matthews C.E. (2019). Association of Leisure-Time Physical Activity Across the Adult Life Course With All-Cause and Cause-Specific Mortality. JAMA Netw. Open.

[161] Matthews C.E., Moore S.C., Arem H. (2020). Amount and Intensity of Leisure-Time Physical Activity and Lower Cancer Risk. J. Clin. Oncol..

[162] Iyengar N.M., Jones L.W. (2019). Development of Exercise as Interception Therapy for Cancer: A Review. JAMA Oncol..

[163] Cabilan C.J., Hines S., Munday J. (2015). The effectiveness of prehabilitation or preoperative exercise for surgical patients: A systematic review. JBI Database Syst. Rev. Implement. Rep..

